# Timing of therapeutic interventions against infection-triggered encephalopathy syndrome: a scoping review of the pediatric literature

**DOI:** 10.3389/fnins.2023.1150868

**Published:** 2023-08-22

**Authors:** Hiroaki Nagase, Hiroshi Yamaguchi, Shoichi Tokumoto, Yusuke Ishida, Kazumi Tomioka, Masahiro Nishiyama, Kandai Nozu, Azusa Maruyama

**Affiliations:** ^1^Department of Pediatrics, Kobe University Graduate School of Medicine, Kobe, Japan; ^2^Department of Neurology, Hyogo Prefectural Kobe Children’s Hospital, Kobe, Japan; ^3^Department of Emergency and General Pediatrics, Hyogo Prefectural Kobe Children’s Hospital, Kobe, Japan

**Keywords:** acute encephalopathy, febrile seizure, status epilepticus, treatment, children

## Abstract

Our goal was to conduct a scoping review of the literature on the treatment of infection-triggered encephalopathy syndrome/acute encephalopathy in children, focusing on treatment targets and treatment initiation timing. We performed literature searches using PubMed for articles reporting treatments of infection-triggered encephalopathy syndrome/acute encephalopathy. We included articles describing specific treatments for acute encephalopathy with control groups. For the purpose of searching new therapies only experimentally tried in the case series, we also included case series studies without control groups in this review, if the studies contained at least two cases with clear treatment goals. Therapies were classified based on their mechanisms of action into brain protection therapy, immunotherapy, and other therapies. We operationally categorized the timing of treatment initiation as T1 (6–12 h), T2 (12–24 h), T3 (24–48 h), and T4 (>48 h) after the onset of seizures and/or impaired consciousness. Thirty articles were included in this review; no randomized control study was found. Eleven retrospective/historical cohort studies and five case–control studies included control groups with or without specific therapies or outcomes. The targeted conditions and treatment timing varied widely across studies. However, the following three points were suggested to be effective in multiple studies: (1) Careful seizure management and targeted temperature management within 12 h (T1) of onset of febrile seizure/prolonged impaired consciousness without multiple organ failure may reduce the development of acute encephalopathy with biphasic seizures and late reduced diffusion; (2) immunotherapy using corticosteroids, tocilizumab, or plasma exchange within 24 h (T1–T2) of onset of acute necrotizing encephalopathy may reduce sequelae; and (3) anakinra therapy and ketogenic diet demonstrate little evidence of neurologic sequelae reduction, but may reduce seizure frequency and allow for weaning from barbiturates, even when administered weeks (T4) after onset in children with febrile infection-related epilepsy syndrome. Although available studies have no solid evidence in the treatment of infection-triggered encephalopathy syndrome/acute encephalopathy, this scoping review lays the groundwork for future prospective clinical trials.

## 1. Introduction

Infection-triggered encephalopathy syndrome (ITES) is characterized by acute encephalopathy (AE). In a national survey in Japan, 34.6% of patients with AE developed mild to severe neurological sequelae (Kasai et al., 2020). AE is a common cause of death in children with ITES. It is defined as syndrome characterized by an acute onset of severe and long-lasting disturbance of consciousness, which typically occurs in previously healthy children (Mizuguchi et al., 2021). In AE, three pathological syndromes interact with each other: excitotoxicity, systemic inflammation/cytokine storm, and metabolic disturbances (Mizuguchi et al., 2007). Excitotoxicity is the main pathological mechanism underlying AE with biphasic seizures and late reduced diffusion (AESD); moreover, systemic inflammation and cytokine storm are associated with acute necrotizing encephalopathy (ANE) and hemorrhagic shock and encephalopathy syndrome (HSES), whereas inflammatory activation in the central nervous system is likely to precede the development of seizures in new-onset refractory status epilepticus (RSE)/febrile infection-related epilepsy syndrome (FIRES) and acute encephalitis with refractory, repetitive partial seizures (AERRPS) (Wickstrom et al., 2022). Specific treatments such as immunotherapy, brain protection treatment, and other treatments have been attempted for AE, taking the abovementioned pathologies into consideration. Moreover, the guidelines for the diagnosis and treatment of AE in childhood recommend the use of corticosteroids, gamma globulin (intravenous immunoglobulin [IVIG]), and cyclosporine for immunotherapy; targeted temperature management (TTM) and osmotic therapy for brain protection; and dextromethorphan, vitamin cocktail therapy, and edaravone for other therapies (Mizuguchi et al., 2021). Real-world surveys have also indicated that these therapies represent treatments of choice (Hayakawa et al., 2020; Murofushi et al., 2022). Making the diagnosis of AE from febrile seizures (FS), especially in patients with AESD, may take several days; hence, the timing and target of each AE treatment modality should be clarified (Yamaguchi et al., 2020; Mizuguchi et al., 2021). Furthermore, the clinical course and final diagnosis can be modified by acute treatment. For example, early intervention for children with febrile status epilepticus may prevent progression to AE (Tokumoto et al., 2022). Notably, interventional studies for AEs vary in terms of the timing of treatment initiation across studies, implying that the purpose of treatment and the implications of treatment efficacy depend not only on the disease condition, but also on the timing of treatment initiation. This poses a challenge for interventional studies. ITES/AE is a rare condition and only a few studies have investigated the efficacy of the abovementioned treatments. Therefore, we conducted a scoping review to systemically map the available research on ITES/AE treatment in children, to determine the extent of the published literature on the efficacy, while focusing on the targeted disease conditions and the timing of treatment initiation.

## 2. Methods

This scoping review was conducted using the PRISMA Extension for Scoping Reviews (PRISMA-ScR): Checklist and Explanation (Tricco et al., 2018).

### 2.1. Search question

The following question was set for this scoping systematic review: What are the effective specific therapies for children with ITES/AE? AE was defined based on the Japanese Society of Child Neurology guidelines for the diagnosis and treatment of AE in childhood (Mizuguchi et al., 2021). The concepts of AE and treatment are shown in Table 1.

**Table 1:** Search strategy.

A. Acute encephalopathy concepts	B. Treatment concepts
Keywords(title/abstract/words)	Keywords(title/abstract/words)
Acute encephalopathy	Treatment
Acute encephalopathy with biphasic seizures and late reduced diffusion	Medication
Acute necrotizing encephalopathy	Therapy
Hemorrhagic shock and encephalopathy syndrome	Intervention
Acute encephalopathy with febrile convulsive status epilepticus	Targeted temperature management
Acute encephalitis with refractory repetitive partial seizures	Vitamins
Febrile infection-related related epilepsy syndrome	Edaravone
Reye syndrome	Cyclosporine
Clinically mild encephalitis/encephalopathy with a reversible splenial lesion	Plasmapheresis
	Anakinra
	Tocilizumab
MeSH terms	MeSH terms
Acute febrile encephalopathy	Time-to-treatment
Hemorrhagic shock and encephalopathy syndrome [Supplementary concept]	Therapeutics
Reye syndrome	Hypothermia, induced
	Immunoglobulins, intravenous
	Immunotherapy
	Mannitol
	Vitamins
	Edaravone
	Cyclosporine
	Plasmapheresis
	Interleukin 1 receptor antagonist protein

Search strategy combination: A and B.

### 2.2. Search strategy

We performed literature searches with PubMed for English articles on the treatment of ITES/AE in humans. Searches were performed independently by two authors, HN and MN, using agreed search terms, and the results were combined. To obtain specific AE treatment or management modalities, we searched the following keywords on May 23, 2023: “((“acute encephalopathy” [All fields] OR “acute encephalopathy with biphasic seizures and late reduced diffusion”[All Fields] OR “acute necrotizing encephalopathy”[All fields] OR “Hemorrhagic shock and encephalopathy syndrome”[All fields] OR “acute encephalopathy with febrile convulsive status epilepticus”[All fields] OR “acute encephalitis with refractory repetitive partial seizures”[All fields] OR “Febrile infection-related epilepsy syndrome”[All fields] OR “Reye syndrome”[All fields] OR “Clinically mild encephalitis/encephalopathy with a reversible splenial lesion”[All fields] OR “Acute febrile encephalopathy”[MeSH terms] OR “Hemorrhagic shock and encephalopathy syndrome”[Supplementary concept] OR “Reye syndrome”[MeSH terms]) AND (“treatment”[All fields] OR “medication”[All fields] OR “therapy”[All fields] OR “intervention”[All fields] OR “Targeted temperature management”[All fields] OR “Vitamins”[All fields] OR “Edaravone”[All fields] OR “Cyclosporine”[All fields] OR “Plasmapheresis”[All fields] OR “Anakinra”[All fields] OR “Time to treatment”[MeSH terms] OR “Therapeutics”[MeSH terms] OR “Hypothermia, induced”[MeSH terms] OR “immunoglobulins, intravenous”[MeSH terms] OR “Immunotherapy”[MeSH terms] OR “Mannitol”[MeSH terms] OR “Vitamins”[MeSH terms] OR “Edaravone”[MeSH terms] OR “Cyclosporine”[MeSH terms] OR “Plasmapheresis”[MeSH terms] OR “Tocilizumab”[All fields] OR “Interleukin 1 receptor antagonist protein”[MeSH terms] OR (“dextromethorphan”[MeSH terms] OR “dextromethorphan”[All fields] OR “dextromethorphane”[All fields])) AND ((casereports[Filter] OR clinicalstudy[Filter] OR clinicaltrial[Filter] OR meta-analysis[Filter] OR observationalstudy[Filter] OR randomizedcontrolledtrial[Filter]) AND (humans[Filter]) AND (allchild[Filter])). Searches were also performed manually.

### 2.3. Study selection

We included articles describing specific treatment modalities for AE with control groups. For the purpose of searching new therapies only experimentally tried in the case series, we also included case series separately from studies with control groups in the review, if they contained two or more cases with clear treatment goals. Eligible articles were identified by performing an initial screen of titles and abstracts, followed by a full review of selected articles. All retrospective and prospective studies that met these criteria were selected.

### 2.4. Data collection

**Figure 1:**
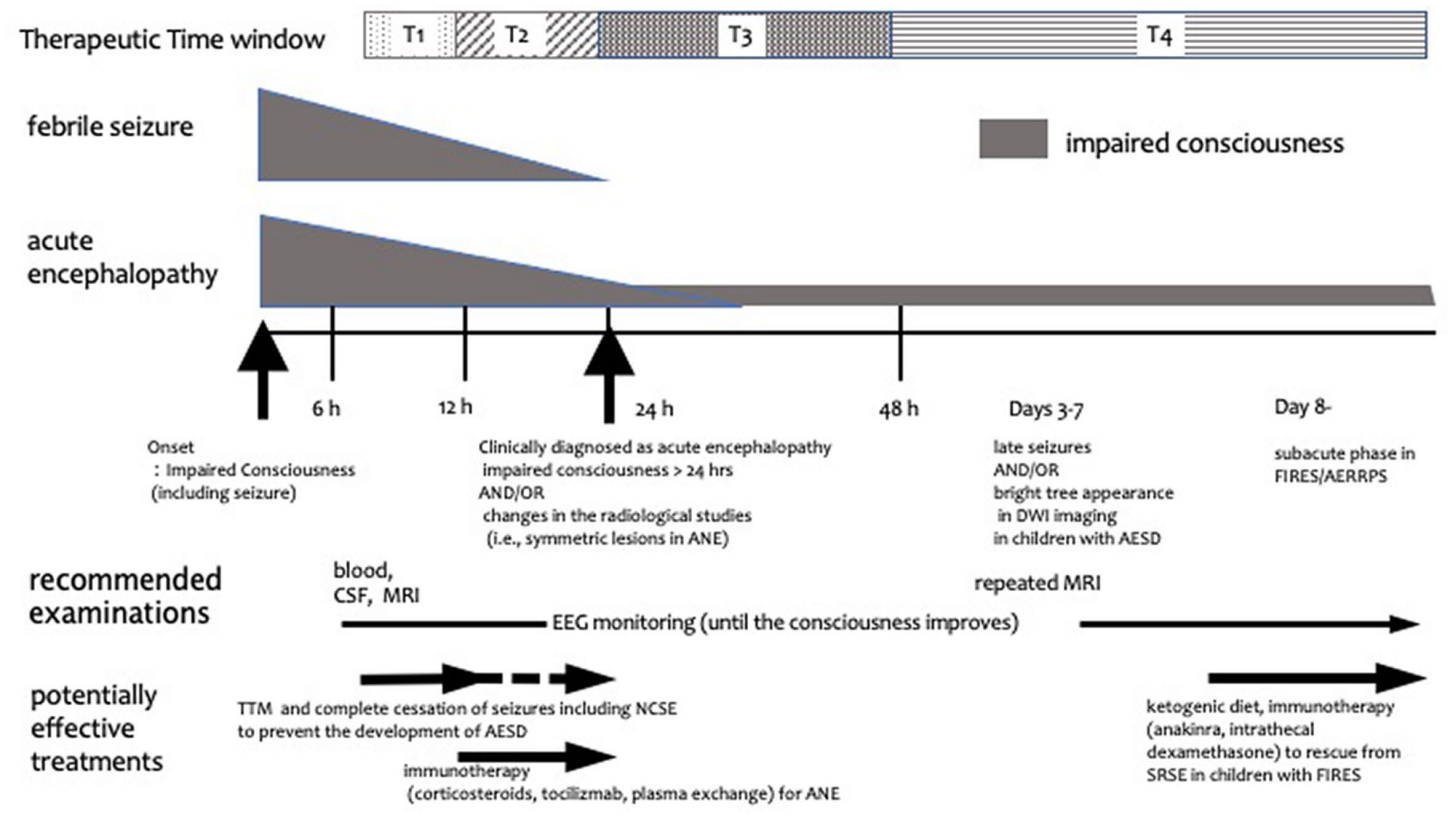
Schematic diagram of treatment timing for acute encephalopathy. Treatment timing is classified into T1, T2, T3, and T4. T1 is the period from 6 to 12 h after the onset of symptoms such as convulsions and impaired consciousness, when there are no imaging abnormalities and the differentiation between febrile seizures and AE has not yet been established. T2 represents the period from 12 to 24 h after onset, whereas T3 is the 24–48 h period after onset, i.e., when AE is diagnosed by prolonged impaired consciousness longer than 24 h and/or radiological findings. T4 is the period after 48 h of onset, i.e., when late seizures or imaging changes of AESD are observed and subacute phase in children with FIRES/AERRPS. The gray shape next to febrile seizure and acute encephalopathy annotations represents the degree and length of seizure and/or impaired consciousness. The horizontal arrows indicate when treatment and examination should be considered. AERRPS, acute encephalitis with refractory, repetitive partial seizures; AESD, acute encephalopathy with biphasic seizures and late reduced diffusion; ANE, acute necrotizing encephalopathy; CSF, cerebrospinal fluid; DWI, diffusion-weighted imaging; EEG, electroencephalogram; FIRES, febrile infection-related epilepsy syndrome; MRI, magnetic resonance imaging; NCSE, non-convulsive status epilepticus; SRSE, super refractory status epilepticus; TTM, targeted temperature management.

The following data were extracted from the selected articles: patient demographics, type of study (retrospective cohort study, case control study, and case series), therapies (target, goal, and timing), outcomes, adverse events, and other therapies. When considering specific treatments for AE; moreover, therapies were classified, based on mechanisms of action, into immunotherapy, brain protection therapy, and other therapies. In addition, it is important to clarify the timing and target of each treatment. In this study, we operationally categorized the timing of treatment initiation as T1–T4 (Figure 1): T1 includes the 6–12 h period after the onset of seizures and/or impaired consciousness, when there are no imaging abnormalities, and differentiation between FS and AE is not yet possible. This timing is consistent with the initiation of neuroprotective treatment for most neurological emergencies (Hypothermia after Cardiac Arrest Study Group, 2002; Shankaran et al., 2005; Hutchison et al., 2008; Hemmen et al., 2010; Legriel et al., 2016). If treatment at this stage is successful without imaging or clinical sequelae, the diagnostic criteria for AE are not fulfilled and the goal of treatment in such a study can be said not to treat AE but to prevent AE development. T2 corresponds to 12–24 h after AE onset, whereas T3 represents 24–48 h after AE onset (i.e., when AE was diagnosed by prolonged impaired consciousness longer than 24 h with or without radiological findings). Finally, T4 corresponds to the period after 48 h of AE onset (i.e., when the definitive diagnosis of AESD was established owing to the manifestation of clustered seizures with or without the characteristic magnetic resonance imaging abnormalities) (Takanashi et al., 2009). The aim of treatment at T3 and T4 was to minimize AE-induced neurological sequelae. Herein, we review comparative studies focusing on the mechanisms and the timing of AE treatment.

## 3. Results

### 3.1. Literature search

**Figure 2:**
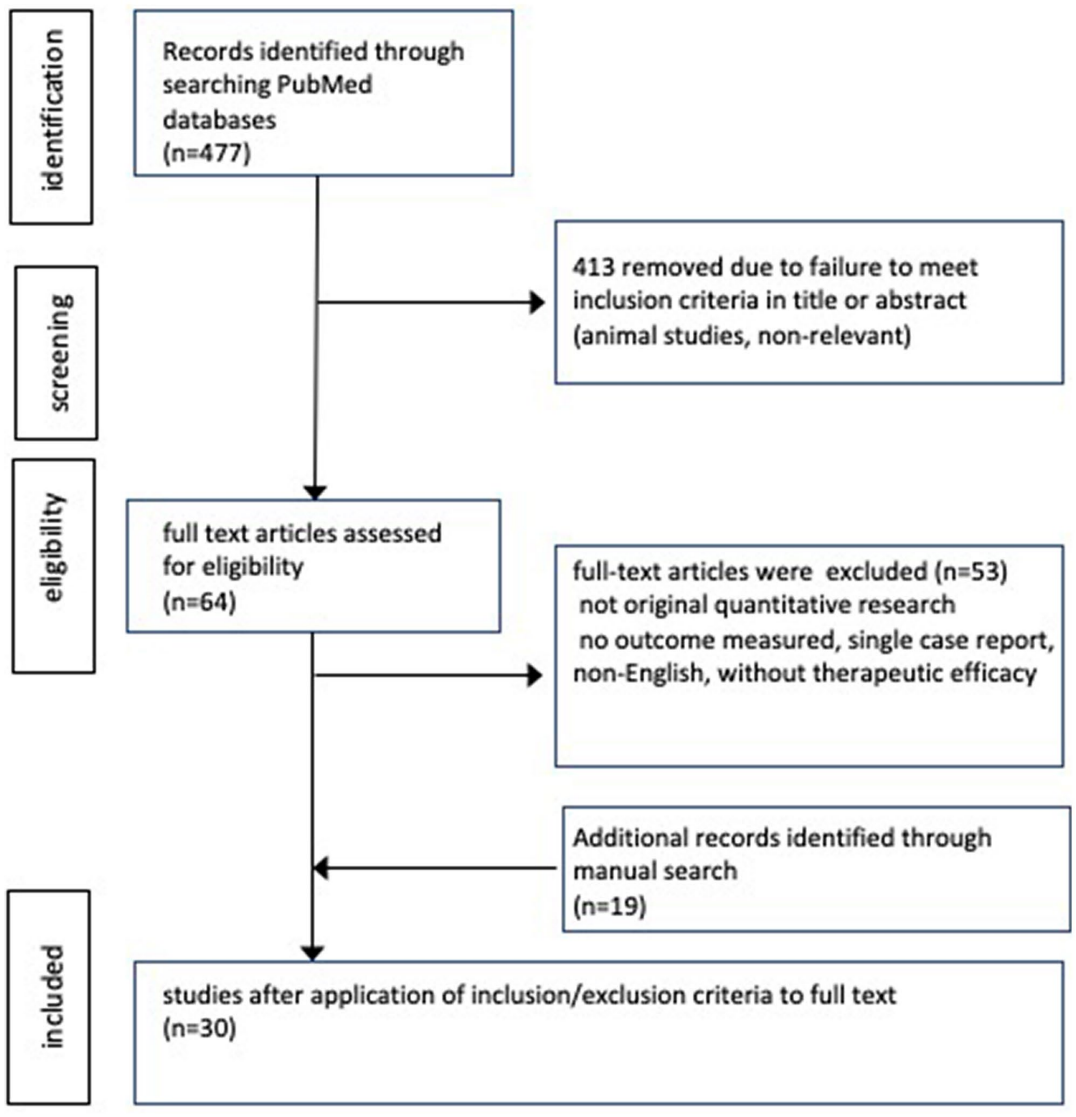
Flow diagram of search strategy.

Figure 2 shows a flow diagram depicting the search results. Overall, 477 articles were identified from PubMed search. After screening titles and abstracts, 64 articles were assessed for eligibility. Subsequently, the eligibility criteria were applied to the full text documents, resulting in the identification of 11 articles from the search; moreover, 19 articles were identified manually through reference screening.

### 3.2. Study characteristics

Thirty articles were included in the review, none of which was a randomized control study. Eleven retrospective/historical cohort studies and five case–control studies included control groups with or without specific therapies or outcomes (Tables 2, 3). Fourteen studies were case series reports without control groups (Tables 4, 5). Among the 16 comparative studies with control groups, 8 studies were on immunotherapies (5, 1, 1, and 1 studies on corticosteroid, plasma exchange [PE], anakinra, and cyclosporine therapies, respectively), 6 studies were on brain protection therapy (5 and 1 studies on TTM [including therapeutic hypothermia] and barbiturate therapy, respectively), and 2 studies were on other treatments, such as vitamin cocktail treatment for mitochondrial dysfunction. Regarding the 14 case series studies, 3 were on brain protection therapy (2 and 1 studies on TTM and pentobarbital therapy, respectively), 10 were on immunotherapies (4, 2, 2, 1, and 1 studies on corticosteroid, tocilizumab, anakinra, cyclosporine, and PE therapies, respectively), and 4 studies were on other therapies (2, 1, and 1 studies on ketogenic diet [KD], centromedian thalamic nuclei deep brain stimulation [CMN-DBS], and dextromethorphan therapy, respectively). The timing of the treatment varied among studies. Multiple studies suggested that the following three strategies were effective: (1) careful seizure management and TTM within 12 h (T1) of onset of FS or prolonged impaired consciousness without multiple organ failure may reduce AESD incidence (Nishiyama et al., 2015; Murata et al., 2016); (2) immunotherapy using corticosteroids or tocilizumab within 24 h (T1–T2) of ANE onset may reduce sequelae (Okumura et al., 2009; Okajima et al., 2022); and (3) treatments such as anakinra therapy and KD demonstrate little evidence of reducing neurologic sequelae, but may reduce seizure frequency and allow for weaning from barbiturates, even when given weeks after onset in children with FIRES. This scoping review laid the groundwork for future prospective clinical trials.

**Table 2:** Retrospective cohort studies and case–control studies including control groups (Study characteristics and patient demographics).

References	Setting	Therapy studied	Category of the therapy	Control	Target of the therapy	Number of patients	Study type/design	Sex (male/female)	Age	Brain stem imaging abnomalities before treatment	Pathogenic bacteria, viruses, and infections
Nishiyama et al. (2015)	Single center	TTM, cooling was initiated within 24 h of onset. Targeted temperature was 34.5 ± 0.5°C or 36.0 ± 0.5°C for at least 48 h. Seven children were treated with the 34.5 ± 0.5°C regimen between October 2002 and December 2005, and 16 children were treated with the 36.0 ± 0.5°C regimen between January 2006 and August 2011.	Brain protection	Conventional care (acetaminophen was usually administered when skin temperature was above 38.5°C.)	Considered to have AE without AST elevation, with inclusion criteria being: (1) seizure with fever (⩾38.0°C) with or without convulsions; (2) RSE and/or prolonged neurological abnormality established by a Glasgow Coma Scale (GCS) score < 15 or the development of hemiplegia 6 h after onset; and (3) a maximum serum AST level within 6 h of onset <90 IU/L.	57 (TTM, *n* = 23; conventional care, *n* = 34)	Retrospective cohort study	TTM 11/12, conventional care 18/16	median (range): TTM 29.0 (8–119) months, conventional care 25.0 (9–159) months	NA	In TTM/conventional care patients: influenza *n* = 6/8, rotavirus *n* = 1/0, human herpes virus 6/7 *n* = 3/1, no specific pathogens *n* = 13/25
Murata et al. (2016)	Single secondary emergency medical care hospital	TTM at 36.0°C 12–72 h and mPSL	Brain protection and immunotherapy	mPSL	Among patients with status epilepticus and fever, those who met any of criteria (1) to (3): (1) Loss or impairment of consciousness with or without other neurological signs persisting over 6 h from presentation, (2) RSE, defined as the persistence of seizure activity despite treatment with at least one antiepileptic drug (3) cases with persistent high voltage slow waves on EEG not responsive to any physical stimulation	20 (TTM/mPSL, *n* = 10; mPSL, *n* = 10)	Retrospective cohort study	TTM/mPSL 7/3, mPSL 5/5	Median (range): TTM/mPSL 17.5 (14–46.8) months, mPSL 17.5 (12.8–30.5) months	NA	In TTM/mPSL / mPSL patients: influenza *n* = 1/2, rotavirus *n* = 2/2, human herpes virus 6 *n* = 3/2, RS virus *n* = 0/1, no specific pathogens *n* = 4/2
Kawano et al. (2011)	10 centers	Therapeutic hypothermia, 33.5–35°C for 48–72 h	Brain protection	Normothermia	AE (ANE 3/5, hemorrhagic shock and encephalopathy syndrome 4/1, AE with refractory seizures 11/5, and others 9/5) in patients with hypothermia/normothermia	43 (hypothermia, *n* = 27; normothermia, *n* = 16)	Retrospective cohort study	Hypothermia 13/14, normothermia 3/13	Median (range): hypothermia 45.0 (25.7–64.4) months, normothermia 29.6 (18.8–40.3) months	NA	In hypothermia/normothermia patients: influenza *n* = 13/4, adenovirus n = 0/1, rotavirus *n* = 1/0, human herpes virus 6/7 *n* = 2/1, others or no preceding infection *n* = 11/10
Sakata et al. (2020)	Single center	Mild hypothermia at 34°C for 3 days (72 h) Eight patients who underwent therapeutic hypothermia administered by an attending physicians before the second phase (late seizure), were included in the Early-Hypo group. Sixteen patients who started therapeutic hypothermia after the initiation of the second phase (late seizure, AE after being diagnosed with AESD) were included in the Late-Hypo group. Ten patients who did not undergo therapeutic hypothermia were included in the Non-Hypo group.	Brain protection	First, differences between the favorable and unfavorable outcome groups were examined, Second, multivariate logistic regression analysis was performed for worse outcomes among patients with AED.	Children who developed or were suspected of having AE. 1. Children with prolonged consciousness disturbance (patients continuously had GCS score < 9) 2. Encephalopathy after cardiac pulmonary arrest and AE associated with infectious diseases. Exclusion criteria: traumatic brain damage, intracranial hemorrhage, unstable vital signs, or severe coagulation disorders	34 patients with acute encephalopathy and reduced subcortical diffusion (AED) (favorable outcome (PCPC<3) *n* = 23, unfavorable outcome (PCPC> = 3) *n* = 11)	Case–control study	Favorable outcome (PCPC <3) 9/14, unfavorable outcome (PCPC ≥3) 4/7	Median (range): favorable outcome (PCPC<3) 24(18–35.5)months, unfavorable outcome (PCPC> = 3) 15(10–18) months	NA	Influenza A *n* = 6, influenza B *n* = 2, HHV6 or exanthem subitem susp *n* = 5, varicella-zoster virus *n* = 2, adenovirus *n* = 1, rotavirus *n* = 1, coxsackievirus A9 *n* = 1, s. pneumoniae meningitis *n* = 1, Kawasaki disease *n* = 1, unidentified *n* = 14
Tokumoto et al. (2022)	Single center	During period I, treatment was administered at the discretion of the attending physician, without a protocol. In clinical practice, FCSE has often been treated with midazolam continuous infusion (MDL ci), without electroencephalogram (EEG) monitoring. There were no standardized rules among physicians regarding the timing of therapeutic interventions in period I. During period II, barbiturate coma therapy (BCT) was indicated for FCSE that was resistant to first-line drugs (mainly benzodiazepines). During period III, BCT was indicated for FCSE resistant to second-line drugs (fos phenytoin or phenobarbital). During periods II and III, thiamylal was mainly used for BCT. During period I, TTM was sometimes implemented at the discretion of the attending physician. During periods II and III, FCSE was treated with TTM when BCT was indicated.	Brain protection	Without protocol-based management	FCSE that persisted for 60 min or longer even after the administration of at least one anticonvulsant (i.e., benzodiazepine). Children diagnosed with encephalitis, such as acute disseminated encephalomyelitis, limbic encephalitis, and pleocytosis in the cerebrospinal fluid (> 8 cells/μL), were excluded. Children with multiple organ failure were also excluded.	110 (period 1 without protocol, *n* = 26; period 2 + 3 with protocol, *n* = 84)	Historical cohort study	Period 1 13/13, period 2 + 3 38/46	median (range): period 1 16.5 (10–29.5) months, period 2 + 3 22 (16, 39.25) months	NA	NA
Hoshide et al. (2020)	Two centers	Therapeutic hypothermia, 34.5 ± 0.5°C for at least 48 h	Brain protection	Historical control without hypothermia therapy	AESD	15 (hypothermia group, *n* = 8; non-hypothermia group, *n* = 7)	Retrospective cohort study	Hypothermia group 3/5, non-hypothermia group 3/4	Median (range): 15.0 (12–21) months in hypothermia group, 16.0 (12–72) months in non-hypothermia group	NA	NA
Kuki et al. (2022)	Single tertiary center	Barbiturate therapy, IVIg, Steroids	Immunotherapy and brain protection	NA	Hemorrhagic shock and encephalopathy syndrome (HSES)	40 (non-death, *n* = 27; death group, *n* = 13)	Case–control study in the single-center case series	16/24	Median (range): 16 months (1 month −15 years)	NA	NA
Ishida et al. (2021)	Single tertiary center	mPSL pulse	Immunotherapy	Non-steroid pulse	Suspected acute encephalopathy in the presence of elevated aspartate aminotransferase (AST) levels (refractory status epilepticus or prolonged neurological abnormality or hemiplegia at 6 h from onset, and AST elevation >90 IU/L within 6 h of onset in children with convulsions or impaired consciousness accompanied by fever [temperature > 38°C])	20 (steroid pulse, *n* = 13; non-steroid pulse, *n* = 7)	Retrospective cohort study	Steroid pulse group 3/10, non-steroid pulse group 6/1	Median (range): 19 (10–62%) months in steroid pulse group, 13(10–19) months in non-steroid pulse group	2 (15%) in steroid pulse group, 1 (14%)in non-steroid pulse group	NA
Okumura et al. (2009)	17 hospitals	Early steroid use (<24 h onset), steroid use, mPSL pulse, IVIg	Immunotherapy	Comparison between patients with good and poor outcome	ANE	34 (with brainstem lesions, *n* = 17; without brainstem lesions, *n* = 17)	Case–control study	With brainstem lesions 7/10, without brainstem lesions 12/5	Median (range): with brainstem lesions 34 (9–86) months, without brainstem lesions 25 (11–67) months	17 cases	In patients with/without brainstem lesions: influenza *n* = 6/5, respiratory illness *n* = 6/8, gastrointestinal illness *n* = 2/3, exanthema subitum *n* = 0/1, not determined *n* = 3/0
Zhu et al. (2021)	Single center	mPSL pulse, IVIg <24 and < 72 h after onset	Immunotherapy	Comparison among groups with mild, moderate, and severe outcome	ANE	41 (mild sequelae, *n* = 8; modetate sequelae, *n* = 12; severe sequelae, *n* = 14)	Case–control study	23/18	Median (interquartile range): 53.9 (8.9–142) months	31 cases	Influenza virus 8, othre etiological infetion 17, no clear etiological infection 16
Hayashi et al. (2012)	Multicenter	Steroid, mPSL pulse, IVIg <48 h after onset and throughout the clinical course	Immunotherapy	Comparison between patients with good and poor outcome	AED	33 patients with acute encephalopathy and reduced subcortical diffusion (AED) (good outcome *n* = 18, poor outcome *n* = 15)	Case–control study	Good outcome 10/8, poor outcome 10/5	Median (range): good outcome 15 (4–40) months poor outcome 25 (8–92) months	NA	Human herpesvirus (HHV)-6 *n* = 8 (24%), HHV-7 *n* = 1 (3%), coxsackie A virus *n* = 2 (6%), influenza A *n* = 1 (3%), mumps in *n* = 1 (3%), enteropathogenic *Escherichia coli n* = 1 (3%). exanthema subitum from their clinical symptoms *n* = 3(9%)
Li et al. (2021)	Four centers	Plasma exchange	Immunotherapy	Non-plasma exhange group	ANE with severity score in plasma exchange/non-plasma exchange median (lower quartile, upper quartile) 2(1,3)/2(0,5)	29 patients with acute necrotizing encephalopathy (ANE)(plasma exchange, *n* = 10; non-plasma exchange, *n* = 19)	Retrospective cohort study	Prescription 15/7, non-prescription 8/4	Median (lower quartile, upper quartile): 30 (19,46) months	In plasma exchange/non-plasma exchange group *n* = 5 (50%)/9 (47.4%)	In plasma exchange/non-plasama exchange group Pathogen positive *n* = 6(60%)/12(63.2%) influenza *n* = 4(40%)/10(52.6%)
Lai et al. (2020)	Multicenter	Anakinra	Immunotherapy	With and without >50% seizure occurrence reduction after 1 week of anakinra treatment.	FIRES	25	Retrospective cohort study	16/9	Median (range): 8 (5.2–11) years	NA	NA
Watanabe et al. (2014)	Single center	Cyclosporine	Immunotherapy	Group A (without cyclosporine)	AESD	14 patients with AESD (without cyclosporine [Group A], *n* = 8; with cyclosporine [GroupB], *n* = 6)	Historical cohort study	Group A 3/5, Group B 2/4	Median (range): Group A 15 (10–32) months, Group B 21.5 (9–31) months	NA	In GroupA/B patients, Influenza *n* = 5/2, Human herpes virus type 6 *n* = 2/2, Epstein–Barr virus *n* = 0/1, unkown *n* = 1/1
Omata et al. (2016)	Single center	Mitochondrial drug cocktail (vitamin B1, vitamin C, vitamin E, biotin, coenzyme Q10, and l-carnitine)	Others (metabolic)	Outcome was compared between prescription and non-prescription groups and between treatment initiation time within 24 h of diagnosis and others (over 24 h or non-prescription)	AE with onset of febrile convulsive status epilepticus (AE was defined based on criteria that have been reported as follows: (a) acute onset of severe and sustained impairment of consciousness after a preceding infection and (b) exclusion of CNS inflammation, in addition to level of consciousness with GCS score ≤ 13 and duration of impairment >24 h, according to the diagnostic criteria of the Japanese research committee on influenza encephalopathy. Febrile convulsive status epilepticus was defined as febrile seizures lasting longer than 30 min.)	21 patients with acute encephalopathy with onset of febrile convulsive status epilepticus (treated with mitochondrial drug cocktail [prescription], *n* = 11; treated within 24 h of diagnosis, *n* = 5; without mitochondrial drug cocktail [non-prescription], *n* = 10)	Retrospective cohort study	Prescription 4/7, non-prescription 3/7	Median (months): prescription 44.4, non-prescription 40.1	NA	In prescription/non-prescription group, Influenza A *n* = 3/1, HHV-6 n = 3/3, Rotavirus *n* = 1/1, Enterovirus *n* = 1/0, unknown *n* = 3/5
Fukui et al. (2019)	Single center	Vitamin B1, vitamin B6, and l-carnitine	Others (metabolic)	Other than prescription group	AE defined as acute onset of severe and sustained impairment of consciousness mostly following an infection. Characteristic clinical features included altered mental status that continued over 24 h (Japan coma scale score > 20; GCS score < 10 or 11), and seizures or focal neurologic signs. Patients with abnormal MRI findings other than bright tree appearance typical for AESD on brain MRI and patients with underlying diseases and/or intervals >24 h between the onset of disease and hospital admission were excluded.	34 patients with AE	Retrospective cohort study	Prescription 15/7, non-prescription 8/4	Median (range): prescription 8 (0–35) months, non-prescription 21 (11–27) months	NA	In prescription/non-prescription group, Influenza *n* = 3/3, HHV-6 *n* = 3/2, HHV-7 *n* = 2/0, RS virus *n* = 3/2, VZV *n* = 1/0, Parecho virus *n* = 1/0, Mycoplasma *n* = 1/0, unknown *n* = 8/5

AE, acute encephalopathy; AED, acute encephalopathy with reduced subcortical diffusion; AESD, acute encephalopathy with biphasic seizures and late reduced diffusion; CNS, central nervous system; EEG, electroencephalogram; ANE, acute necrotizing encephalopathy; FCSE, febrile convulsive status epilepticus; FIRES, febrile infection-related epilepsy syndrome; IV, intravenous; Ig, immunoglobulin; mPSL, methylprednisolone; NA, not applicable; PCPC, pediatric cerebral performance category; RSE, refractory status epilepticus; TTM, targeted temperature management.

**Table 3:** Retrospective cohort studies and case control studies including control (group therapies, response, outcome, adverse effects therapies for AE/ITES, response, outcome, adverse effects).

References	Therapy studied	Therapy category	Control	Therapy target	Therapy goal	Outcome evaluation	Timing of the therapy (classification)	Duration from neurological symptom onset to therapy initiation	Other anti-convulsive medications	Other immunotherapies	Other brain protection therapies	Other therapies	Patient outcome	Adverse effects	Final diagnosis
Nishiyama et al. (2015)	TTM, cooling was initiated within 24 h of onset. Targeted temperature was 34.5 ± 0.5°C or 36.0 ± 0.5°C for at least 48 h. Seven children were treated with the 34.5 ± 0.5°C regimen between October 2002 and December 2005, and 16 children were treated with the 36.0 ± 0.5°C regimen between January 2006 and August 2011.	Brain protection	Conventional care (acetaminophen was usually administered when skin temperature was above 38.5°C.)	Considered to have AE without AST elevation, with inclusion criteria being: (1) seizure with fever (⩾38.0°C) with or without convulsions; (2) RSE and/or prolonged neurological abnormality established by a Glasgow Coma Scale (GCS) score < 15 or the development of hemiplegia 6 h after onset; and (3) a maximum serum AST level within 6 h of onset <90 IU/L.	Neurological outcome	Neurological performance at 1 month after onset of acute encephalopathy was assessed using the PCPC Scale	T1-2	Within 24 h of onset	Diazepam 17/30, midazolam iv 21/27, phenobarbital sodium iv 4/2, midazolam continuous iv 11/19, thiamylal continuous iv 20/4 in TTM/conventional care patients	Steroids 7/3, steroid pulse 2/0, immunoglobulin 0/0 in TTM/conventional care patients	Mannitol 11/20 in TTM/conventional care patients	Tracheal intubation 23/8, inotropic support 22 / 5 in TTM/conventional care patients. During cooling and rewarming, all children were intubated and anesthetized intravenously, with continuous administration of anticonvulsants and muscle relaxants in TTM patients.	Good patient outcomes (PCPC = 1) were observed in 23/23 (100%) and 23/34 (70.6%) in the TTM and conventional care groups, respectively (p = 0.004)	No severe adverse events occurred in either group during the intervention period.	No patient fufilled the diagnostic criteria of acute encephalopathy syndrome in the TTM group. Acute encephalopathy with febrile convulsive status epilepticus (currently referred to AESD, including hemiconvulsion–hemiplegia syndrome) 8 cases, acute encephalitis with refractory, repetitive partial seizures 1 case, and heat stroke 1 case in the conventional care group.
Murata et al. (2016)	TTM at 36.0°C 12–72 h and mPSL	Immunotherapy and brain protection	mPSL	Among patients with status epilepticus and fever, those who met any of criteria (1) to (3): (1) Loss or impairment of consciousness with or without other neurological signs persisting over 6 h from presentation, (2) RSE, defined as the persistence of seizure activity despite treatment with at least one antiepileptic drug (3) Cases with persistent high voltage slow waves on EEG not responsive to any physical stimulation	Neurological outcome	Neurologic performance 6 months after onset was evaluated using the PCPC scale. Patients were categorized into the poor prognosis group when the PCPC score increased after the onset of AE or when there were obvious neurological sequelae even if the PCPC score had not increased	T1	TTM therapy was initiated within 8 h of onset of convulsions. Time from the onset of convulsion to start of the specific treatment (h),TTM/mPSL group 5.5(4–7), mPSL group 4.5 (4–7.8)	Administered anticonvulsants to stop the seizures based on the recommendation of the Epilepsy Foundation of America’s Working Group on status epilepticus and the Japanese guidelines. Cases were monitored by EEG, and complete seizure cessation was confirmed both clinically and by absence of electrographic seizure activity on EEG.	No	No	Ventilator management and muscle relaxant therapy were used only for severe respiratory failure.	Prognosis at 6 months after onset: good prognosis (PCPC = 1) in 10/10 (100%) and 6/10 (60%) patients in TTM/mPSL and mPSL groups, respectively (p = 0.0433)	No	0 (0%) and 4 (40%) patients were finally diagnosed with AESD in the TTM/mPSL and mPSL groups, respectively. The other patients were diagnosed with non-AESD acute encephalopathy.
Kawano et al. (2011)	Therapeutic hypothermia, 33.5–35°C for 48–72 h	Brain protection	Normothermia	AE (ANE 3/5, hemorrhagic shock and encephalopathy syndrome 4/1, AE with refractory seizures 11/5, and others 9/5) in patients with hypothermia/normothermia	Neurological outcome	PCPC score at 12 months from the development of encephalopathy/encephalitis	T1-2	16.7 (10.3–23.0) h	Thiopental (up to 3 mg/kg/h) or midazolam (up to 1 mg/kg/h) unless contraindicated	Steroids 24/10, IVIg 14/4 in hypothermia/normothermia patients	NA	Intravenous vecronium or pancronium (up to 0.1 mg/kg/h) was considered when shivering was uncontrollable.	Good outcome (PCPC ≤3) was associated with early hypothermia initiation (<12 h) (p = 0.004)	Hypotension 11/7, pneumonia 9/2, thrombocytopenia 12/9, coagulation disorder 9/7, hypolkalemia 16/10 in hypothermia/normothermia patients	NA
Sakata et al. (2020)	Mild hypothermia at 34°C for 3 days (72 h) Eight patients who underwent therapeutic hypothermia by an attending physicians before the second phase (late seizure), were included in the Early-Hypo group. Sixteen patients who started therapeutic hypothermia after the initiation of the second phase (late seizure, AE after being diagnosed with AESD) were included in the Late-Hypo group. Ten patients who were not exposed to therapeutic hypothermia were included in the Non-Hypo group.	Brain protection	First, the differences between the favorable and unfavorable outcome groups were examined. Second, multivariate logistic regression analysis was performed for worse outcomes among patients with AED.	Children who developed or were suspected of having AE. 1. Children with prolonged consciousness disturbance (patients continuously had GCS score < 9) 2. Encephalopathy after cardiac pulmonary arrest and AE associated with infectious diseases. Exclusion criteria: traumatic brain damage, intracranial hemorrhage, unstable vital signs, or severe coagulation disorders	Neurological outcome	Neurological outcomes at 12 months from disease onset were assessed using the PCPC score. PCPC scores of 1or 2 and those of 3–6 were classified as favorable and unfavorable outcomes, respectively.	T1-4	Before and after the second phase of acute encephalopathy with reduced subcortical diffusion in the Early-Hypo (T1-3) and Late-Hypo (T4) groups, respectively.	Continuous midazolam infusion was administered in two patients. Thyamylal infusion was administered for the remaining patients during therapeutic hypothermia. Dexmedetomidine was administered in eight patients Midazolam 0.1–0.6 mg/kg/h Thiamyral/thiopental 1.0–3.0 mg/kg/h Dexmedetomidine 0.2–0.9 μg/kg/h	IV mPSL pulse or IVIg were used at the discretion of the attending physicians before February 2017; however, authors refrained from using these two therapies for patients with AED since March 2017, except for one patient with Kawasaki disease, accoding to the revision of Japanese practice guideline for acute encephalopathy in childhood in 2016. In total, mPSLwas used in 15 patients and IVIg was used in 8 patients.	Head-up position of 5–30° for patients with severe brain swelling	All patients with therapeutic hypothermia were intubated and mechanically ventilated. Mechanical ventilator management: FiO2 ≤ 0.6, PaO2 > 100–150 torr (mmHg), PCO2 = 35–45 torr (mmHg) (avoid hyperventilation), respiratory rehabilitation Muscle relaxant for intractable shivering: Vecronium 0.06 mg/kg/h Analgesia: fentanyl 1–3 μg/kg/h. Maintain blood pressure to keep cerebral perfusion pressure (= mean blood pressure - ICP) > 50 mmHg Continuous EEG monitoring	The unfavorable group included more patients with therapeutic hypothermia, although the between-group difference was not statistically significant (*p* = 0.113). Moreover, there were no between-group differences in clinical variables including IVmPSL, IVIg administration, or dexmedetomidine use during cooling. A strong association was found between the worse outcomes and the Time1st-cooling (*p* = 0.004), and the duration from the first seizure to the time when the body temperature reached 35°C (Time1st-35°C) (*p* = 0.002) or 34°C (Time1st-34°C) (p = 0.004) in the Early-Hypo group (T1-3). There was no association between the outcomes and the Time1st-cooling in the overall AED population with therapeutic hypothermia (Early-Hypo plus Late-Hypo groups T1-4) (*p* = 0.270). Additionally, no association was observed between the outcomes and the Time2nd-cooling (*p* = 0.440), and the duration between the first seizure to the time when the body temperature reached 35°C (Time2nd-35°C, *p* = 0.665) or 34°C (Time2nd-34°C, *p* = 0.366) in the Late-Hypo group (T4).	In favorable outcome (PCPC<3) /unfavorable outcome (PCPC> = 3), hypothension 7 (30.4)/3 (27.2), Catecholamine use, n (%) 8 (34.8) /9 (81.8) (*p* = 0.026), pneumonia 3 (13.0)/4 (36.4), thrombocytopenia, n (%) 9 (39.1)/9 (91.8) (*p* = 0.003), coagulation disorder, n (%) 16 (69.6)/10 (90.9), arrhythmia, n (%) 0 (0%)/0 (0%), hypokalemia <3.5 mEq/L, n (%) 15 (65,2)/10 (90.9)	NA
Tokumoto et al. (2022)	During period I, treatment was administered at the discretion of the attending physician, without a protocol. In practice, FCSE has often been treated with midazolam continuous infusion (MDL ci), without electroencephalogram (EEG) monitoring. There were no standardized rules among physicians regarding the timing of therapeutic interventions in period I. During period II, barbiturate coma therapy (BCT) was indicated for FCSE that was resistant to first-line drugs (mainly benzodiazepines). During period III, BCT was indicated for FCSE resistant to second-line drugs (fPHT or PB). During period II and III, thiamylal was mainly used for BCT. During period I, TTM was sometimes implemented at the discretion of the attending physician. During periods II and III, FCSE was treated with TTM when BCT was indicated.	Brain protection	Without protocol-based management	FCSE that persisted for 60 min or longer even after the administration of at least one anticonvulsant (i.e., benzodiazepine). Children diagnosed with encephalitis, such as acute disseminated encephalomyelitis, limbic encephalitis, and pleocytosis in the cerebrospinal fluid (> 8 cells/μL), were excluded. Children with multiple organ failure were also excluded.	Neurological outcome and development of acute encephalopathy	PCPC scale score at the time of the last medical examination, within 90 days after onset. PCPC score ≥ 2 was defined as a poor outcome.	T1	Time from onset to BCT initiation (h): period 1 5.5 (3.9, 37.1), period 2 + 3 6.8 (5.0, 9.0)	*n*(%), diazepam 25 (96.2)/74 (88.1), midazolam iv 19 (73.1)/69 (82.1), fos phenytoin iv 0 (0)/23 (27.4)(p < 0.001), phenobarbital 9 (34.6)/15 (17.9), midazolam continuous iv 22 (84.6)/21 (25.0)(*p* < 0.001), thiamylal continuous iv 9 (34.6)/38 (45.2) in period 1/period 2 + 3 patients	No	TTM (target 34°C or 36°C) with ventilation, muscle relaxants, and vasopressors, as needed. Continuous EEG monitoring was also performed during BCT. TTM n(%), 11 (42.3)/38 (45.2), EEG monitoring 3 (11.5)/ 72 (85.7) (*p* < 0.001) in period 1/period 2 + 3 patients	Vasopressor, n (%) 13 (50.0)/37 (44.0) in period 1/period 2 + 3 patients	Pediatric Cerebral Performance Category (PCPC) Scores ≥2, n(%): 6 (23.1)/6 (7.1) (*p* = 0.03), in period 1/period 2 + 3 patients	Ventilator-associated pneumonia, n (%) 0 (0)/7 (8.3) in period 1/period 2 + 3 patients	*n*(%) acute encephalopathy (AE) 9 (34.6)/11 (13.1) (p = 0.02), acute encephalopathy with biphasic seizures and late reduced diffusion (AESD) 5 (19.2)/6 (7.1), clinically mild encephalitis/encephalopathy with a reversible splenial lesion (MERS) 0 (0)/1 (1.2), unclassified AE 4 (15.4)/4 (4.8), febrile seizure 17 (65.4)/73 (86.9) (*p* = 0.02)
Hoshide et al. (2020)	Therapeutic hypothermia, 34.5 ± 0.5°C for at least 48 h	Brain protection	Historical control without hypothermia therapy	AESD	Neurological outcome, mental disability, and debvelopment of post-encephalopathic epilepsy (PEE)	Neurological performance at 1 month after the onset of AESD assessed using the PCPC score. Mental disability was assesed using the Wechsler Intelligence Scale for Children (≥7 years), Tanaka-Binet Intelligence Scale (≥3 years), and Enjoji Infantile Developmental Scale (<3 years). PEE was defined as multiple seizures that occurred 14 days after first onset of the illness that required continuous use of anticonvulsants.	T4	Within 24 h after the onset of late seizure (days 3–7)	Anti-seizure medication (hypothermia vs. non-hypothermia), midazolam div. (0,3), thiopental div, iv (8,4), phenobarbital div (6,3), lidocaine div (0,2), edaravone div (8,5)	Hypothermia vs. non-hypothermia: intravenous immunoglobulin and/or intravenous methylprednisolone (1,4)	(hypothermia vs. non-hypothermia): edaravone (8,5)	No	Tha number of patients who had PCPC score (1,2,3,4,5,6) in hypothermia (4,2,2,0,0,0), in non-hypothermia (3,1,2,1,0,0) (*p* = 0.5362), mental disability (normal, border, mild, moderate, severe) in hypothermia (6,0,1,1,0), in non-hypothermia (3,0,2,0,2) (*p* = 0.5362), PEE (non, focal epilepsy) in hypothermia (8,0), in non-hypothermia (3,4) (*p* = 0.0256)	NA	NA
Kuki et al. (2022)	Barubiturate therapy, IVIg, Steroids	Immunotherapy and brain protection	NA	Hemorrhagic shock and encephalopathy syndrome (HSES)	Neurological outcome	Survive or death	NA	NA	NA	Cyclosporine *n* = 11 (27.5%)	Brain hypothermia therapy *n* = 27 (67.5%), edaravone n = 13 (32.5%)	Blood purification therapy, *n* = 7 (17.5%)	Non-death group vs. death group, univariate analysis: barbiturate therapy (85.2% vs. 46.2%; OR, 0.149 [95% CI, 0.033–0.683]; *p* = 0.014), IVIg (66.7% vs. 30.8%; OR, 0.222 [95% CI, 0.054–0.923]; p = 0.038), IV mPSL (85.2% vs. 46.2%; OR, 0.149 [95% CI, 0.033–0.683]; *p* = 0.014). Multivariate analysis was not performed.	NA	NA
Ishida et al. (2021)	mPSL pulse	Immunotherapy	Non-steroid pulse	Suspected acute encephalopathy in the presence of elevated aspartate aminotransferase (AST) levels (refractory status epilepticus or prolonged neurological abnormality or hemiplegia at 6 h from onset, and AST elevation >90 IU/L within 6 h of onset in children with convulsions or impaired consciousness accompanied by fever [temperature > 38°C])	Neurological outcome	PCPC score of 1–2 at the last evaluation, within 30 months of onset.	T1-2	<24 h	Median number of anti seizure medications are 3(2–3) in steroid pulse group, 4(2.5–4) in non steroid pulse group	No	TTM 9/13 in steroid pulse group, 5/7 in non steroid pulse group	Mitochondrial rescue drugs (vitamin cocktail) 5 (38%) in steroid pulse group, 0(0%) in non steroid pulse group	Good outcome (PCPC = 1–2) was achieved in 5(38%) and 0 (0%) patients in the steroid pulse and non-steroid pulse groups, respectively.	NA	AE 10 (77%), cytokine storm type 3 (23%), AESD 4 (31%), unclassified 3 (23%), febrile seizure 3 (23%) in steroid pulse group AE 5 (71%), cytokine storm type 3 (43%), AESD 1 (14%), unclassified 1 (14%), febrile seizure 2 (29%) in non-steroid pulse group
Okumura et al. (2009)	Early steroid therapy (<24 h of onset), steroid use, mPSL pulse, IVIg	Immunotherapy	Comparison between patients with good and poor outcomes	ANE	Neurological outcome	Good outcome (intelligence or development quotient 50 ≥ without motor impairment) and poor outcome	T1-2, T1-4	Early steroid administration (within 24 h of ANE onset), steroid use, steroid pulse therapy throughout the clinical course	Antiepileptic drugs were administered in all patients.	No	NA	NA	Significant differences were found between early steroid use (good outcome 7/12) and late steroid use (good outcome 0/5) in patients without brain stem lesions (p = 0.044). The outcome of patients was not correlated with steroid use, steroid pulse therapy, or gammaglobulin.	No	NA
Zhu et al. (2021)	mPSL pulse, IVIg <24 and < 72 h after onset	Immunotherapy	Comparison among groups with mild, moderate, and severe outcomes	ANE	Neurological outcome	Outcome of surviving children was determined 1 year or longer after the onset of ANE based on both cognitive and motor impairments. Cognitive impairment was categorized as normal (DQ/IQ ≥ 70), mild (50 ≤ DQ/IQ < 70), moderate (30 ≤ DQ/IQ < 50), and severe (DQ/IQ < 30) according to the Gesell Developmental Scales (applicable to children <6 years) or Wechsler Intelligence Test (applicable to children ≥6 years). Motor impairment was categorized as normal, mild (walking with or without support), moderate (sitting with or without support), and severe (required support to sit and stand).	T1-2, T1-4	Categolized before and after 24 and 72 h after onset of ANE	NA	NA	Hypothermia 24 (59%)	NA	There was no significant association between early steroid and IVIg therapy (within 24 or 72 h after onset) and neurological sequelae. 7 patients (17%) died, and the remaining 34 survivors had different degrees of neurological sequelae, predominantly moderate and severe types (mild, 24%; moderate, 35%; severe, 41%)	NA	NA
Hayashi et al. (2012)	Steroid therapy, mPSL pulse, IVIg <48 h after onset and throughout the clinical course	Immunotherapy	Comparison between patients with good and poor outcomes	AED	Neurological outcome	Trained pediatric neurologists judged the neurodevelopmental outcome as good when the patient had non-existent or mild cognitive and/or mild motor impairment, and patient outcomes were judged as poor when patients had more severe neurologic impairment.	T1-3, T1-4	Categorized before 48 h after onset	Twenty-five children (76%) were treated with antiepileptic drugs such as diazepam, phenobarbital, midazolam, or phenytoin within 48 h after the onset. None of our patients underwent high-dose barbiturate therapy.	No	No patient received therapeutic hypothermia.	Mechanical ventilation was administered in 0 (0%) and 4 (27%) in good and poor outcome patients.	IVIg was more commonly used in children with poor outcomes than in those with good outcomes. Treatment with steroids and/or IVIg was unrelated to good outcomes.	NA	NA
Li et al. (2021)	Plasma exchange	Immunotherapy	Non-plasma exhange group	ANE with severity score in plasma exchange/non-plasma exchange median (lower quartile, upper quartile) 2(1,3)/2(0,5)	Mortality	Died at discharge or at the last follow up	T1-4	From consciousness disturbance to the first plasma exchange cycle was 32 h (range, 6–78 h). in 8(80%) patients within 24 h after the admission to the PICU	NA	In plasma exchange/non-plasma exchange group: steroids *n* = 10(100%)/18(94.7%), intravenous immunoglobulin *n* = 10(100%)/18(94.7%)	NA	In plasma exchange/non-plasama exchange group: mechanical ventilation *n* = 9(90%)/13(68.4%), antiviral therapy *n* = 10(100%)/17(89.5%)	Plasma exchange/non-plasma exchange groups, n (%) or median (lower quartile, upper quartile) Duration of PICU stay (days) 10.1(2.8, 19.1)/3.9 (2.0, 13.0) Length of stay (days) 16.9 (3.5, 23.1)/8.0 (2.0, 19.0) Died at discharge *n* = 0 (0%)/9(47.4%) (*p* = 0.011) Died at the last follow-up *n* = 3 (33.3)/10 (58.8) (*p* = 0.411)	NA	NA
Lai et al. (2020)	Anakinra	Immunotherapy	With and without >50% seizure occurrence reduction at 1 week of anakinra treatment.	FIRES	Neurological outcome and seizure reduction	The Pediatric Cerebral Performance Category (PCPC) was determined by the site investigators. Neuropsychological domain assessments were performed by the respective contributing centers and reported as normal or abnormal. In a subset of children (*n* = 15), local investigators determined electrographic and electroclinical seizure frequency immediately before and 1 week after anakinra treatment. Subsequently, authors dichotomized these children into those with >50% seizure reduction (*n* = 11) and those without seizure reduction (*n* = 4). They evaluated the demographics, clinical characteristics, and outcomes using descriptive statistics. Pearson correlation was used to evaluate the association between the timing of anakinra initiation and duration of mechanical ventilation, ICU length of stay (LOS) and hospital LOS	T4	Median of 20 days [14–25 days] after seizure onset.	Two children were on midazolam infusion alone; 5 on pentobarbital alone; and 18 on both infusions. All children received at least 4 additional anti-seizure medications (ASMs), with 18 children (72%) having experienced failure to at least 7 medications prior to anakinra initiation. Nineteen children (76%) were on the ketogenic diet; 7 (28%) received cannabinoids.	Corticosteroids and intravenous immunoglobulin (IVIG) were used in 22 children (88%), 11 (44%) and 5 (20%) of whom received plasmapheresis and rituximab, respectively.	NA	All the patients received mechanical ventilation	Earlier anakinra initiation after seizure onset was associated with shorter duration of mechanical ventilation, ICU LOS, and hospital LOS (*r* = 0.46 [*p* = 0.03], *r* = 0.50 [*p* = 0.01], and *r* = 0.48 [*p* = 0.03], respectively). Among children with available seizure frequency data (*n* = 15), 11 exhibited >50% seizure occurrence reduction at 1 week of anakinra treatment. The median interval between seizure onset and anakinra initiation was 19 (12–30) and 27 (13.5–37.5) days in children with and without seizure occurrence reduction. The median duration of mechanical ventilation and ICU LOS were 35.5 (22–44) days and 47.5 (34–108) days in children with seizure reduction; 50.5 (35.5–111.5) days and 66 (43.5–70) days in children without seizure occurrence reduction.	Nine children (36%) had infections prior to anakinra initiation whereas 10 (40%) had infections following treatment (Table 2). Three children (12%) developed drug reaction with eosinophilia and systemic symptoms (DRESS) syndrome, which was treated with the addition or escalation of corticosteroids. All patients recovered without complications. Two children (8%) developed cytopenias that eventually resolved without specific intervention. Anakinra was discontinued in one child due to infection.	NA
Watanabe et al. (2014)	Cyclosporine	Immunotherapy	GroupA (without cyclosporine)	AESD	Neurodevelopmental outcome	Assessed at 6 months from disease onset. As prognostic measures, we used the PCPC and the Pediatric Overall Performance Category scale (POPC)	T4	The period from late seizure onset (3–6 days of illness) to AESD treatment initiation ranged from 1 to 24 h (median, 10.5 h in group A; 16 h in group B; *p* = 0.173).	NA	In GroupA (without cyclosporine)/ Group B (with cyclosporine): methylprednisolone pulse *n* = 7/6, i.v immnoglobulin *n* = 0/0	In Group A (without cyclosporine)/ B (with cyclosporine): edaravone *n* = 7/6, hypothermia *n* = 2/0	NA	No significant difference in PCPC score between groups A and B in the total population (*p* = 0.072). Significant between-group difference was found when patients with frontal lobe-predominant lesions were excluded(*p* = 0.020).	Three patients experienced hypertension due to cyclosporine use.	NA
Omata et al. (2016)	Mitochondrial drug cocktail (vitamin B1, vitatimin C, vitamin E, biotin, coenzyme Q10, and l-carnitine)	Others (metabolic)	Outcome was compared between prescription and non-prescription groups, and between treatment initiation time within 24 h of diagnosis and others (over 24 h or non-prescription)	AE with onset of febrile convulsive status epilepticus (AE was defined based on criteria that have been reported as follows: (a) acute onset of severe and sustained impairment of consciousness after a preceding infection and (b) exclusion of CNS inflammation, in addition to level of consciousness with GCS score ≤ 13 and duration of impairment >24 h, according to the diagnostic criteria of the Japanese research committee on influenza encephalopathy. Febrile convulsive status epilepticus was defined as febrile seizures lasting longer than 30 min.)	Neurological outcome	Patients were classified in terms of sequelae into (A) no sequelae group (having no obvious functional decline in motor or intelligence function) or (B) sequelae group (all others). The family and attending physician determined the presence or absence of sequelae at the time of hospital discharge.	T1-4, T1-3	Treated throughout the clinical course or 24 h of diagnosis (within 48 h of onset)	NA	Steroid pulse therapy was started within 24 h of encephalopathy diagnosis in all the 21 patients	NA	NA	No significant difference in the distribution of the severity of sequelae (A vs. B) between the mitochondrial drug cocktail prescription and non-prescription groups in all 21 patients and in both AESD and monophasic groups. Sequelae were significantly better in the group that received the drug cocktail within 24 h of diagnosis (within 24 h of onset) compared with other groups (over 24 h or non-prescription) (*p* = 0.035)	NA	AESD *n* = 11, monophasic *n* = 10
Fukui et al. (2019)	Vitamin B1, vitamin B6 and l-carnitine	Others (metabolic)	Other than prescription group	AE defined as acute onset of severe and sustained impairment of consciousness mostly following an infection. Characteristic clinical features included altered mental status that continued over 24 h (Japan coma scale score > 20; GCS score < 10 or 11), and seizures or focal neurologic signs. Patients with abnormal MRI findings other than bright tree appearance typical for AESD on brain MRI and patients with underlying diseases and/or intervals >24 h between the onset of disease and hospital admission were excluded.	Neurological outcome and development of acute encephalopathy	Developmental delay and development of epilepsy. Development of AESD assessed at the last visit at 4.5 (2–11) and 7.0 (2–11) years of age in the prescription and non-prescription groups	T1-2	Within 24 h of onset	NA	Both groups received the same glucocorticoid pulse therapy regimen	Both groups received the same edaravone regimen	Intubation was performed in 16 (73%) and 10 (83%) patients in prescription and non-prescription groups.	Prescription/non-prescription groups: Developmental delay, *n* = 1 (5%)/4(36%) (*p* = 0.037), development of epilepsy, *n* = 1 (5%)/5(45%) (*p* = 0.011), development of AESD, *n* = 2 (9%)/7(58%) (*p* = 0.004)	NA	In prescription/non-prescription patients, AESD *n* = 2/7, mild encephalopathy (without a second AESD attack such as late seizrure) *n* = 20/5

AE, acute encephalopathy; AED, acute encephalopathy with reduced subcortical diffusion; AESD, acute encephalopathy with biphasic seizures and late reduced diffusion; CNS; central nervous system; EEG, electroencephalogram; ANE, acute necrotizing encephalopathy; FCSE, febrile convulsive status epilepticus; FIRES, febrile infection-related epilepsy syndrome; IV, intravenous; Ig, immunoglobulin; mPSL, methylprednisolone; NA, not applicable; PCPC, pediatric cerebral performance category; RSE, refractory status epilepticus; TTM, targeted temperature management.

**Table 4:** Case series studies without control groups (pediatric study characteristics and patient demographics).

References	Setting	Therapy studied	Therapy category	Therapy target	Number of patients	Study type/design	Sex (male/female)	Age	Brain stem imaging abnormalities before treatment	Pathogenic bacteria, viruses, and infection
Lin et al. (2012)	Single center	Therapeutic hypothermia, 33°C	Brain protection and immunotherapy	SRSE in patients with FIRES	2 cases	Case series	1/1	4, 10 years	NA	No pathogen was detected in patient 1, NA in patient 2
Boutros et al. (1977)	Single center	Therapeutic hypothermia, 31°C	Brain protection	Reye syndrome (all patients with clinical stage 4 and EEG grade 4 or worse)	5 cases	Case series	4/1	2–14 years	NA	NA
Marshall et al. (1978)	Single center	Pentobarbital	Brain protection	Reye syndrome (three patients with EEG stage 4)	7 cases	Case series	3/4	0.8–17 years	NA	Influenza B *n* = 3, Coxackie B *n* = 1, Varicella *n* = 1, negative *n* = 3
Seo et al. (2010)	Single center	IV dexamethasone or mPSL pulse therapy	Immunotherapy	ANE	6 cases	Case series	3/3	1–7 years	5 cases	Non-specific febrile illness *n* = 4, upper respiratory tract infection *n* = 2, diarrhea *n* = 1
Lee et al. (2014)	Single center	Steroid pulse and IVIg therapy	Immunotherapy	ANE	11 epidodes in 10 patients	Case series	5/5	Median (range), 1.5 years (0.4–8.4 years)	7 episodes	Influenza A *n* = 2, influenza A(H1N1) *n* = 1, influenza B *n* = 1, parainfluenza *n* = 2, rotavirus *n* = 1, none *n* = 4
Okajima et al. (2022)	Single center	Intravenous methylprednisolone and therapeutic plasma exchange (TPE)	Immunotherapy	ANE with a severity score of 3, one case ANE with severity score of 8, two cases	2 cases	Case series	2/0	3, 7 years	NA	Case 1: H1N1pdm2009 influenza virus. Case 2: H3Nx influenza virus
Horino et al. (2021)	Single center	Intrathecal dexamethasone therapy (IT-DEX)	Immunotherapy	FIRES	6 cases	Case series	5/1	3–6 years	NA	NA
Shrestha et al. (2023)	Multicenter	Anakinra	Immunotherapy	FIRES	6 cases	Case series	NA	11.08 years (interquartile range 8.19–11.23 years) at status epilepticus onset	NA	NA
Sa et al. (2019)	Single center	Centromedian thalamic nuclei deep brain stimulation (CMN-DBS) and anakinra treatment	Immunotherapy and others	SRSE in patients with FIRES	2 cases	Case series	2/0	5, 7 years	NA	NA
Matsuo et al. (2013)	Single center	Dextromethorphan and cyclosporine A	Immunotherapy and others	AESD in four cases, mild consciousness disturbance 24 h after the first prolonged febrile seizure (near-miss AESD) in four cases	Four cases with AESD and four cases with near-miss AESD (Patients who showed mild consciousness disturbance 24 h after the first prolonged febrile seizure and recovered without having secondary seizures)	Case series	5/3	8–18 months	NA	Exanthema subitum *n* = 7, unknown *n* = 1
Koh et al. (2019)	Single center	Tocilizumab	Immunotherapy	ANE (all patients had an ANE severity score of 5)	3 cases	Case series	1/2	5.1, 10.3, 8.0 years	All three patients	Influenza A(H1N1)pdm09 *n* = 2, influenza B *n* = 1
Hosie et al. (2023)	Single center	Tocilizumab	Immunotherapy	ANE with a severity score of 6, one case ANE with severity score of 5, two cases	2 cases	Case series	2/0	19 months, 3 years	2 cases	Case 1: noro virus, Case 2: influenza A (type H3), adenovirus, and rhinovirus
Nabbout et al. (2010)	Five centers	Ketogenic diet	Others	SRSE in patients with FIRES	9 cases	Case series	4/5	54–98 months (mean 74 months)	NA	Pharyngitis *n* = 4, flu *n* = 1, gastroenteritis *n* = 1, vaccination *n* = 2, unknown *n* = 1
Singh et al. (2014)	Single center	Ketogenic diet	Others	SRSE in patients with FIRES	2 cases	Case series	1/1	7, 10 years	NA	streptococcal pharyngitis *n* = 1, unknown *n* = 1

AESD, acute encephalopathy with biphasic seizures and late reduced diffusion; EEG, electroencephalogram; ANE, acute necrotizing encephalopathy; FIRES, febrile infection-related epilepsy syndrome; IV, intravenous; Ig, immunoglobulin; mPSL, methylprednisolone; NA, not applicable; SRSE, super refractory status epilepticus.

**Table 5:** Case series studies without control group (therapies, response, outcome, adverse effects).

References	Therapy studied	Therapy category	Therapy target	Therapy goal	Outcome evaluation	Timing of the therapy (classification)	Duration from neurological symptom onset to therapy initiation	Other anti-convulsive medications	Other immunotherapies	Other brain protection therapies	Other therapies	Patient outcome	Adverse effects	Final diagnosis
Lin et al. (2012)	Therapeutic hypothermia, 33°C	Brain protection	SRSE in patients with FIRES	Termination of SRSE	Clinical and EEG evaluation	T1	8 and 12 h after admission	IV lorazepam/diazepam, followed by phenytoin, phenobarbital, valproic acid, and midazolam	IVIg and high-dose IVmPSL	Head elevation, hyperosmotic agents (mannitol and hypertonic saline), and hyperventilation	IV acyclovir and oral oseltamivir	Clinical and electrographical seizures were terminated in both patients	Patient 1, transient hypokalemia	NA
Boutros et al. (1977)	Therapeutic hypothermia, 31°C	Brain protection	Reye syndrome (all patients with clinical stage 4 and EEG grade 4 or worse)	Neurological outcome	Clinical evaluation	NA	Immediately after admission	NA	Dexamethasone	Mannitol	Fluid restriction, neomycin, vitamin K, full curarization, mechanical ventilation, intracranial pressure monitoring, and perctaneous brachial arterial cannulation	Complete recovery of four patients; one patient died.	NA	NA
Marshall et al. (1978)	Pentobarbital	Brain protection	Reye syndrome (three patients with EEG stage 4)	Intracranial pressure (ICP) control	Amount of mannitol necessary to control ICP	T1	From day 1 (when ICP > 30 mm Hg for at least 30 min)	NA	Steroids	Mannitol and hyperventilation	No	The mean (± SEM) mannitol requirement before barbiturate administration and on the day after attainment of a satisfactory blood barbiturate level were 3.7 ± 0.3 g/kg/day and 0.5 ± 0.2 g/kg/day (*p* < 0.001), respectively.	NA	NA
Seo et al. (2010)	IV dexamethasone or mPSL pulse therapy	Immunotherapy	ANE	Neurological outcome	Clinical evaluation at 6 months	NA	NA	NA	No	NA	NA	Excellant, complete resolution *n* = 1; Good, almost complete resolution or minimal degree of neurological sequelae (mobile, almost no cognitive/social/emotional impairment) *n* = 1; Fair, moderate degree of neurological sequelae (mobile with difficulty, mild to moderate cognitive/social/emotional impairment) n = 2; Poor, severe degree of neurological sequelae (immobile, severe cognitive/social/emotional impairment) *n* = 2.	No	NA
Lee et al. (2014)	Steroid pulse and IVIg therapy	Immunotherapy	ANE	Neurological outcome	Clinical evaluation	T1-3	Within 24 h of ANE diagnosis	NA	No	NA	Treatment for possible viral encephalitis or bacterial meningitis such as vancomycin, and third-generation cephalosporin, or acyclovir and oseltamivir for influenza A- or B-positive patients.	Death *n* = 4, developmental language delay *n* = 2, normal *n* = 4, epilepsy *n* = 2, mortor deficit *n* = 1, transient motor deficit *n* = 1	NA	NA
Okajima et al. (2022)	Intravenous methylprednisolone and therapeutic plasma exchange (TPE)	Immunotherapy	ANE with a severity score of 3, one case ANE with severity score of 8, two cases	Neurological outcome	Clinical evaluation	For mmPSL T1, for TPE T1-2	Case 1: IV mPSL and TPE with continuous hemodiafiltration at 8 h and 16 h after onset, Case 2: IV mPSL administered 5 h after onset; underwent TPE with continuous hemofiltration at 9 h after onset.	Fosphenytoin, levetiracetum	NA	Strictly controlled to normal deep body temperature, edaravone, and mannitol	Vitamins	Both patients showed normal cognitive and behavioral development on the 3-year follow-up visit.	NA	NA
Horino et al. (2021)	Intrathecal dexamethasone therapy (IT-DEX)	Immunotherapy	FIRES	EEG changes, termination of SRSE, cognitive outcome	Changes in EEG findings. Duration from IT-DEX initiation to thiopental discontinuance, duration of mechanical ventilation, and adverse events. Cognitive outcomes and seizure frequency at hospital discharge.	T4	Days 9,10,10,20,20,59	Four of the six patients needed sustained thiopental to control seizures at the time of initiation of IT-DEX treatment. The remaining two patients showed SE persistence, despite burst-suppression coma.	Among the patients, corticosteroid and IVIg therapy were administered in 22 children (88%), of whom 11 (44%) underwent plasmapheresis and 5 (20%) used rituximab. Four to 11 anti-seizure medication were administered before IT-DEX.	No	All patients received mechanical ventilation	Six patients were withdrawn from anesthetic agents, after a median of 5.5 days from the initiation of IT-DEX. Seizure frequency decreased after IT-DEX administration in three patients (50%). Seizure foci were localized and background activities improved in all patients. There was a positive correlation between the length of stay in the ICU and the duration from the disease onset to the introduction of IT-DEX (rs = 0.85, *p* = 0.03) as well as the duration of mechanical ventilation (rs = 0.88, *p* = 0.03). All patients, except one, showed PCPC score > 3 at hospital discharge.	No	NA
Shrestha et al. (2023)	Anakinra	Immunotherapy	FIRES	Neurocognitive outcomes, seizure burden	Median follow-up of 40 months (IQR 35–51 months)	T4	11.0 days (IQR 9.25–13.50)	During the acute phase, all patients received pentobarbital treatment and obtained burst-suppression on EEG. Five patients received pentobarbital treatment during the acute period. Two patients also underwent neuromodulation (vagus nerve stimulation or responsive neurostimulation).	All patients received IVIg treatment, and five patients received corticosteroids.	NA	All patients continued to take a median of 3.5 (IQR 2–5) daily anti-seizure medications at the time of their last neuropsychological evaluation. Two patients also underwent neuromodulation (vagus nerve stimulation or responsive neurostimulation).	Seizure burden before anakinra administration was 4 episodes per week as well as 18,22,42,52, and 100 per day. Seizure burden at hospital discharge were 0, 1, 1, 2 per month as well as 1–2 per week. All patients had ongoing seizures and no patient returned to baseline cognitive function, with a median follow-up of 40 (IQR 35–51) months. Of the five patients with serial full-scale IQ scores, three demonstrated a decline in scores over time.	NA	NA
Sa et al. (2019)	Centromedian thalamic nuclei deep brain stimulation (CMN-DBS) and anakinra treatment	Immunotherapy and others	SRSE in patients with FIRES	Termination of SRSE, neurological outcome	Clinical evaluation	T4	Patient 1: CMN-DBS was started on day27,Anakinra was started on D43 Patient 2: CMN-DBS was started on day 37 and anakinra was started on day 22	Phenobarbitone, midazolam, thiopentone, ketamine, ketogenic diet, and cannabidiol	No	No	No	CMN-DBS abolished generalized seizures in both cases. Patient 1 had good neourological outcome (attended mainstream class) and patient 2 had poor outcome (vegetative state).	No	NA
Matsuo et al. (2013)	Dextromethorphan and cyclosporine A	Immunotherapy and others	AESD in four cases, mild consciousness disturbance 24 h after the first prolonged febrile seizure (near-miss AESD) in four cases	Neurological outcome	The evaluation of motor and cognitive development was performed using the Enjoji Scale of Analytical Development for Infant and Toddler	In AESD cases T4, in near-miss AESD cases T3-4	In patients with AESD, dextromethorphan and cyclosporine A were started when the secondary seizure was observed on days 4–6. In patients with near-miss AESD, dextromethorphan was started on days 2–3	Diazepam, midazolam, phenobarbital, thiamylal, and carbamazepine	No	No	No	Of four patients with AESD, three were normal after 1–3 years. Hyperactivity was observed in one patient after 3 years. Of four patients with near-miss AESD, no patients showed late seizures. Neurological outcome was not reported.	No	NA
Koh et al. (2019)	Tocilizumab	Immunotherapy	ANE (all patients had an ANE severity score of 5)	Neurological outcome	Modified Rankin Scale	T2-3	18, 32, and 20 h (<24 h after the ANE diagnosis)	NA	High dose IV mPSL in three cases, IVIg in one case	NA	IV Ceftriaxone, IV Acyclovir, Oral Oseltamivir	Outcome (mRS score), timing 0, at 1 month follow-up 0, at 2 months follow-up 2, at 3 months follow-up	No	NA
Hosie et al. (2023)	Tocilizumab	Immunotherapy	ANE with a severity score of 6, one case ANE with severity score of 5, two cases	Long-term neurological outcome	Modified Rankin Scale	T2, T4	Case 1: tocilizumab at 53 h, 1 week, and 4 week Case 2: tocilizumab at 22 h and 1 week	Case 2: levetiracetam	Case 1: IVIg, mPSL at 21 and 39 h, Case 2: dexamethasone, mPSL, and intravenous IVIg at 1, 21, 22 h	NA	Case 1: anti-epileptic and anti-microbial therapy. Both patients were intubated and ventilated	Case 1: Profound cortical visual impairment, spastic-dystonic quadriplegic cerebral palsy, and bulbar dysfunction. His modified Rankin scale (mRS) score at discharge was 5 and remains unchanged after 2 years. Case 2: Severe dyskinetic quadriplegia, visual impairment, expressive aphasia, and cerebellar dysfunction. mRS scores at discharge and 2 years later were 4 and 3, respectively.	NA	NA
Nabbout et al. (2010)	Ketogenic diet	Others	SRSE in patients with FIRES	Termination of SRSE	Seizure termination	T4	Days 4–55	Number of anti-epileptic drugs 3 to 6, pentobarbital *n* = 6	Steroids	NA	NA	Seizures stopped in seven patients, within 2–4 days (mean 2 days) following the onset of ketonuria and 4–6 days (mean 4.8 days) following the onset of the ketogenic diet.	NA	NA
Singh et al. (2014)	Ketogenic diet	Others	SRSE in patients with FIRES	Termination of SRSE	Clinical evaluation	T4	3 and 13 hospitalization days	Patient 1: phenytoin, phenobarbital, levetiracetam, valproic acid, and topiramate as well as continuous infusions of midazolam and pentobarbital Patient 2: fosphenytoin, phenobarbital, levetiracetam, lacosamide, topiramate, valproic acid, lorazepam, and pentobarbital	IV mPSL	No	No	Both patients could not be weaned off pentobarbial.	No	NA

AESD, acute encephalopathy with biphasic seizures and late reduced diffusion; EEG, electroencephalogram; ANE, acute necrotizing encephalopathy; FIRES, febrile infection-related epilepsy syndrome; IV, intravenous; Ig, immunoglobulin; mPSL, methylprednisolone; NA, not applicable; SRSE, super refractory status epilepticus.

## 4. Discussion

Since the 1970s, many AE treatment options have been reported in patients with Reye syndrome; however, this scoping review revealed limited evidence for AE treatment efficacy. This review found no randomized control trial evaluating the AE treatment efficacy; only 16 retrospective cohort and case–control studies with control groups as well as 14 case series reports of at least two patients were found. In the present study, we classified the treatment options into brain protection therapy, immunotherapy, and other therapies, based on the mechanism of action of each treatment; moreover, we categorized the timing of treatment initiation after neurological symptom onset as follows: T1 (6–12 h), T2 (12–24 h), T3 (24–48 h), and T4 (>48 h). The timing of treatment initiation, target condition (confirmed diagnoses of AE or high risk of developing AE), and treatment goal (improvement of neurological outcome and freedom from status epilepticus) differed significantly among the studies. We have hereinafter discussed each treatment option based on the mechanism of action, timing of initiation, and target conditions.

### 4.1. Brain protection

#### 4.1.1. TTM

Therapeutic hypothermia is theoretically effective for various brain injuries, and its mechanisms of action include reduction of oxygen and nutrient demand, suppression of glutamate and free radical production, suppression of apoptosis, suppression of inflammatory cytokine production, suppression of brain edema, and anticonvulsant effects (Polderman, 2008). A recent study showed no difference between therapeutic hypothermia with TTM at 33°C and a control intervention with TTM at 36°C (Nielsen et al., 2013). To maintain the body temperature at 36°C and avoid hyperthermia is now included in the notion of TTM at normothermia. Two studies have reported AESD incidence reduction and fewer sequelae in patients who underwent TTM within 24 h after onset (T1–T2) compared with patients who did not (Nishiyama et al., 2015; Murata et al., 2016). Nishiyama et al. conducted a retrospective cohort study on TTM at 34.5 ± 0.5°C or 36.0 ± 0.5°C for at least 48 h within 24 h of AE onset (T1–T2) in children at high risk of developing AE-induced neurological sequelae (Nishiyama et al., 2015). In the TTM group, most children were maintained in a hypothermic state until 12 h of onset (T1). They found that 23/23 (100%) and 23/34 (70.6%) of children had good outcomes in the TTM and conventional care groups, respectively (*p* = 0.004). Eight children with sequelae in the conventional care group fulfilled the diagnostic criteria for AESD, whereas no child without sequelae developed AESD. Murata et al. conducted a historical (retrospective) cohort study on children who underwent TTM at 36.0°C for 12–72 h combined with methylprednisolone (MP) use (TTM/MP) (Murata et al., 2016). For children who developed febrile status epilepticus that was clinically diagnosed as AE, TTM therapy was initiated within 8 h of seizure onset (T1). The conventional treatment group received only MP treatment, and the prognosis at 6 months after onset was good (PCPC = 1) in 10/10 (100%) and 6/10 (60%) patients in the TTM/MP and MP groups, respectively (*p* = 0.0433). No child (0%) and 4 (40%) children were finally diagnosed as having AESD in the TTM/MP and MP groups, respectively. TTM has a narrow time window; hence, the targeted temperature was reached within 12 h of the onset of impaired consciousness (T1) in many patients in the abovementioned two studies. Kawano et al. (2011) reported that the induction of TTM after 12 h (T2–T4) of AE onset (including final diagnoses of ANE [*n* = 3/5], HSES [*n* = 4/1], AE with refractory seizures [*n* = 11/5], and others [*n* = 9/5] in patients with hypothermia/normothermia) did not improve outcomes. In a case–control study, Sakata et al. (2020) examined the time to TTM initiation in children having AE with reduced subcortical diffusion (including AESD) with or without neurological sequelae and in those without late seizures. They found that the time from onset of status epilepticus to TTM induction correlated with the outcome at T1, whereas the time from onset of late seizures at T4 to TTM initiation did not correlate with the outcome. Tokumoto et al. (2022) reported the hourly protocol orient management including TTM aiming to start treatment around 6 h after onset (T1) for febrile convulsive status epilepticus reduced neurological sequelae and cases finally diagnosed as AE even though the ratio who underwent TTM were not different. Hoshide et al. (2020) conducted a historical cohort study involving the use of therapeutic hypothermia in children with identified late seizures and a diagnosis of AESD confirmed at T4. They found that the frequency of neurologic sequelae did not differ between the therapeutic hypothermia and conventional treatment groups; nevertheless, the development of post-encephalopathic epilepsy was significantly lower in the therapeutic hypothermia group. Two case series reports have been published on this subject. Lin et al. (2012) reported two cases of patients with FIRES who developed super RSE (SRSE) after admission, which was successfully treated via therapeutic hypothermia at 33°C at 8 h and 12 h after admission (T1). In Boutros et al. (1977) reported a case series of Reye syndrome. Although no specific timing was provided, they performed protocol-based management according to the intracranial pressure (ICP) immediately after admission, including therapeutic hypothermia at 31°C, volume control, and rescue treatment using mannitol. Four of five patients with clinical stage IV Reye syndrome (estimated fatality rate at that time, 50–100%) recovered without sequelae; however, one patient died. In these reports, status epilepticus in the TTM group was assessed using electroencephalographic (EEG) monitoring; moreover, all the reports included patients undergoing immune therapy such as corticosteroid therapy, and hence the impact of such supportive care should be considered. Therefore, although the standard timing for TTM in patients with AE has not yet been established, TTM remains the only effective treatment for AE as reported in multiple comparative studies; furthermore, it seems more appropriate to administer TTM within 12 h of AE onset (T1) to reduce neurological sequelae and AESD development.

#### 4.1.2. Barbital therapy

Marshall et al. (1978) reported a case series wherein pentobarbital was administered to reduce ICP in seven patients with Reye syndrome, who were classified as stage IV using EEG classification. Phenobarbital was administered when ICP was greater than 30 mmHg for at least 30 min (T1). Pentobarbital administration reduced the amount of mannitol per day required for ICP control from 3.7 ± 0.3 g/kg/day to 0.5 ± 0.2 g/kg/day (*p* < 0.001). Kuki et al. (2022) conducted a case–control study in patients with HSES in their institution. Univariate analysis comparing between non-death and death groups revealed that barbiturate therapy (85.2% vs. 46.2%; odds ratio [OR], 0.149 [95% confidence interval [CI], 0.033–0.683]; *p* = 0.014) as well as IVIG (66.7% vs. 30.8%; odds ratio [OR], 0.222 [95% confidence interval [CI], 0.054–0.923]; *p* = 0.038) and intravenous MP therapies (85.2% vs. 46.2%; odds ratio [OR], 0.149 [95% confidence interval [CI], 0.033–0.683]; *p* = 0.014) were significantly more applied in the non-death group; however, they did not describe the timing of barbiturate initiation.

### 4.2. Immunotherapy

#### 4.2.1. Corticosteroid therapy

Corticosteroid therapy is theoretically effective for AE when the primary pathophysiology is systemic inflammatory response and cytokine storm. Since its recommendation in the Japanese influenza encephalopathy guideline (Morishima, 2006), it has been the most commonly used specific treatment for AE in Japan (Hayakawa et al., 2020). However, only a few comparative studies have investigated the efficacy of corticosteroid therapy. To our knowledge, only our previous study has hitherto compared the use and non-use of corticosteroids in children suspected to be at high risk of inflammation-mediated AE (Ishida et al., 2021). We used corticosteroid pulse therapy in children with aspartate aminotransferase levels >90 U/L within 6 h after AE onset, in whom the suspected pathophysiological mechanism was systemic inflammatory response or cytokine storm, based on our clinical prediction rule (Nagase et al., 2013; Sasaki et al., 2017). In our retrospective cohort study, we investigated the efficacy of corticosteroid pulse therapy within 24 h of AE onset (T1–T2) in children at high risk of cytokine storm-mediated AE (Ishida et al., 2021). The study revealed no difference in the neurological sequelae; good outcome (PCPC = 1–2) was achieved in 5 (38%) and 0 (0%) patients in the corticosteroid and non-corticosteroid pulse groups, respectively. However, there was a trend toward better outcomes in patients who underwent earlier the treatment initiation within several hours of onset (T1) (Ishida et al., 2021). Furthermore, three case–control studies have compared the proportions of corticosteroid-treated children with and without neurological sequelae (Okumura et al., 2009; Hayashi et al., 2012; Zhu et al., 2021). Okumura et al. (2009) conducted a multi-center case–control study on patients with ANE and reported that patients who underwent early corticosteroid therapy (MP pulse or dexamethasone administration) within 24 h (T1–T2) for ANE without brainstem lesions developed fewer moderate or severe sequelae (5 of 12 patients) than those without early corticosteroid therapy (5 of 5 patients) (*p* = 0.044); nevertheless, the outcome of patients was not correlated with corticosteroid use, corticosteroid pulse therapy, or gammaglobulin use throughout the clinical course at T1–T4. Zhu et al. (2021) conducted a case–control study in children with and without ANE-induced neurological sequelae. They found no difference in the efficacies of corticosteroid pulse therapy and intravenous gammaglobulin use within the periods <24 h (T1–T2) and < 72 h (T1–T4) after onset between groups (Zhu et al., 2021). Hayashi et al. (2012) reported that corticosteroid therapy within 48 h of onset (T1–T3), or during the entire period of late seizures (T1–T4), was not associated with outcome in patients with AESD (Hayashi et al., 2012). In a case–control study of 21 patients with AE associated with *E. coli* O111 infection, who were classified according to neurological prognosis, Takanashi et al. reported that corticosteroid pulse therapy was associated with prognosis; however, this study was not included in the current analysis because 11 of the 21 participants were adults (Takanashi et al., 2014). Case series studies of corticosteroid therapy without control groups included a study suggesting that patients with ANE who received corticosteroid pulse therapy instead of dexamethasone had fewer sequelae (timing was not indicated) (Seo et al., 2010); a study showing that corticosteroid pulse therapy with IVIG within 24 h of ANE diagnosis (T1–T3) was not associated with mortality (Lee et al., 2014); and a report of two cases of children with ANE treated by intravenous MP administered 5 and 8 h after onset (T1), combined with therapeutic PE and TTM at normothermia, both of whom completely recovered (Okajima et al., 2022). Horino et al. (2021) evaluated the efficacy of corticosteroid use in patients with AEs who underwent intrathecal dexamethasone therapy (IT-DEX) for FIRES. IT-DEX was administered on days 9–59 of FIRES (T4), and all six patients were weaned from anesthesia within a median of 5.5 days after IT-DEX administration. Seizure frequency decreased in 3 (50%) patients after IT-DEX administration. Seizure foci became more localized and background activity improved in all patients. The duration of intensive care unit (ICU) stay was positively correlated with the time from onset to IT-DEX administration (rs = 0.85, *p* = 0.03) and the duration of ventilation (rs = 0.88, *p* = 0.03). Therefore, although corticosteroid therapy is theoretically effective for AEs, the disease conditions, disease severity, and timing of treatment that result in clinical efficacy have not been sufficiently clarified. In our studies on the dynamics of inflammatory cytokines in patients with HSES (a cytokine storm-mediated AE) and ICU-treated patients with febrile status epilepticus, inflammatory cytokine levels peaked within 24 h after neurological symptom onset, followed by a gradual decline in cytokine levels (Yamaguchi et al., 2021; Tomioka et al., 2023). Probably, corticosteroids should be administered preferably within 12 h (T1) and at most within 24 h (T1–T2) of onset to improve the outcome of patients with cytokine storm-mediated AEs associated with systemic inflammation, such as ANEs. For conditions such as AERRPS/FIRES, in which cerebral inflammation is a possible pathogenesis, interventions performed later (T4), such as weaning from RSE, may be effective.

#### 4.2.2. IVIG therapy

IVIG therapy is thought to have an anti-inflammatory action via the suppression of immunocompetent cell activation and inflammatory cytokine production. It is theoretically reasonable to use IVIG in patients with inflammation-associated AE, such as cytokine storm-mediated AE. Thus, IVIG is recommended in AE management guidelines (Mizuguchi et al., 2021) and is the second most used AE treatment after corticosteroid therapy (Hayakawa et al., 2020). However, only one comparative study has demonstrated the efficacy of IVIG. In a case–control study of patients with HSES, Kuki et al. (2022) reported an association of neurological outcome with IVIG use as well as corticosteroid therapy and barbiturate use (non-death group vs. death group, 66.7% vs. 30.8%, respectively; OR, 0.222 [95% CI, 0.054–0.923]; *p* = 0.038); nevertheless, multivariate analysis showed no significant association. Furthermore, the following three studies have compared outcomes with and without IVIG use in patients with AE: IVIG use for ANE was not associated with outcome (good prognosis in 2 of 5 patients exposed to IVIG use and in 5 of 12 patients without IVIG use) (T1–T4) (Okumura et al., 2009); IVIG use for ANE within 24 h (T1–T2) and within 72 h (T1–T4) were not associated with outcome (Zhu et al., 2021); and IVIG use for AESD within 48 h of onset (T1–T3) (IVIG use: 1 of 18 patients had good outcomes, and 2 of 15 patients had poor outcomes, *p* = 0.43) or during the entire course (T1–T4) (IVIG use: 3 of 18 patients had good outcomes, and 9 of 15 patients had poor outcomes, *p* = 0.010) did not effectively improve outcomes (Hayashi et al., 2012).

In a case series study, Lee et al. (2014) demonstrated that IVIG with corticosteroid pulse therapy within 24 h of ANE diagnosis (T1–T3) was not associated with mortality.

#### 4.2.3. PE therapy

PE is also theoretically effective against cytokine storm because of its ability to rapidly remove inflammatory substances from the blood. Li et al. (2021) conducted a retrospective comparative study to investigate the efficacy of PE therapy in patients with ANE. The outcomes of 10 pediatric ICU (PICU)-admitted patients who underwent PE was compared with those of 19 patients without PE. The duration from disturbance of consciousness to the first cycle of PE ranged from 6 h to 78 h, with a median of 32 h (T1–T4), and in 8 (80%) patients consciousness disturbance continued within 24 h (T1–T2) after PICU admission until the first cycle of PE. In the non-PE group, 18/19 children also underwent corticosteroid and IVIG therapy. In-hospital deaths were clearly lower in the PE (0%) than the non-PE (47.4%) group (*p* = 0.011); however, the mortality rate at the final follow-up was not significantly different between the two groups (33.3 and 58.8% for the PE and non-PE groups, respectively, *p* = 0.411). The improvement in liver enzymes was more rapid in the PE group. In a case report, two children with ANE were treated using intravenous MP combined with therapeutic PE (which started at 9 h and 16 h (T1–T2) from the onset) and TTM at normothermia, both of whom completely recovered (Okajima et al., 2022).

#### 4.2.4. Anakinra

Recent studies have suggested that neuroinflammation may be involved in the pathogenesis of FIRES/AERRPS (Clarkson et al., 2019). Lai et al. (2020) conducted an international retrospective cohort study of 25 children treated with anakinra, a recombinant interleukin (IL)-1 receptor antagonist, as an immunomodulator for FIRES. Earlier anakinra initiation after seizure onset was associated with shorter durations of mechanical ventilation (*r* = 0.46, *p* = 0.03) as well as ICU (*r* = 0.50, *p* = 0.01) and hospital length of stay (*r* = 0.48, *p* = 0.03). Among children with available seizure frequency data (*n* = 15), 11 exhibited >50% seizure occurrence reduction at 1 week of anakinra treatment. Although there were no statistically significant differences due to the small sample size, the median interval between seizure onset and anakinra initiation tended to be shorter in children with seizure occurrence reduction (19 days [range, 12–30 days]) than in those without seizure occurrence reduction (27 days [range, 13.5–37.5 days]) (T4). Nevertheless, there was no such tendency in terms of neurological outcomes at discharge (Lai et al., 2020). Shrestha et al. reported a case series of six children with FIRES who underwent anakinra treatment at a median of 11 days of illness (T4). Their seizure burden, which was >10 episodes per day prior to anakinra treatment, reduced to <1–2 episodes per week after anakinra treatment initiation. However, none of the patients returned to their baseline cognitive function, and the median follow-up duration was 40 months (interquartile range, 35–51 months). Of the five patients with serial full-scale intelligence quotient scores, three demonstrated a decline in scores over time (Shrestha et al., 2023). Sa et al. reported two cases of patients with FIRES treated with CMN-DBS and anakinra. In patient 1, CMN-DBS and anakinra treatments were initiated on days 27 and 43 (T4), respectively. In patient 2, CMN-DBS and anakinra treatments were started on days 37 and 22 (T4), respectively. CMN-DBS abolished the occurrence of generalized seizures in both patients. The neurological outcome of patient 1 was good, as the patient was able to attend a mainstream class, whereas patient 2 had a poor outcome, as the patient was in a vegetative state (Sa et al., 2019).

#### 4.2.5. Cyclosporine

Cyclosporine use can inhibit hypercytokinemia-associated apoptosis and prevent cytotoxicity and tissue damage progressions. Watanabe et al. (2014) reported the efficacy of cyclosporine use in children with AESD without frontal lobe-predominant damage after late seizures (T4). Three of five cyclosporine-treated patients had good prognosis, and two patients had mild sequelae; however, two and three of five non-cyclosporine-treated patients had mild and moderate sequelae, respectively (*p* = 0.020) (Watanabe et al., 2014). Matsuo et al. reported cases of four patients with AESD treated with cyclosporine and dextromethorphan, an N-methyl-D-aspartate (NMDA) receptor antagonist, after the occurrence of late seizures (T4) (Matsuo et al., 2013). Three patients recovered without sequelae, whereas one patient had mild sequelae. Although the number of patients was small, it is noteworthy that cyclosporine treatment was administered after the occurrence of late seizures (T4) in both studies.

#### 4.2.6. Tocilizumab

In patients with ANE, brain injury is presumed to result from a cytokine storm; moreover, these patients reportedly have increased blood IL-6 levels in the acute phase (Kawamura et al., 2013). Only two case series reports on the use of tocilizumab, a monoclonal antibody against the IL-6 receptor, for ANE treatment are hitherto available. Koh et al. reported cases of three patients with ANE who received tocilizumab at 18–32 h of onset (T2–T3). Two of the patients recovered without sequelae, and one patient developed mild sequelae (the modified Rankin Scale [mRS] score was 2) (Koh et al., 2019). Hosie et al. reported two ANE cases. The patient in case 1 received IVIG, MP, and tocilizumab at 21, 39, and 53 h of onset (T4 for tocilizumab), respectively. His mRS at discharge was 5 and remained unchanged 2 years later. The patient in case 2 received dexamethasone, MP, and tocilizumab at 1, 21, and 22 h, (T2 for tocilizumab), respectively. Researchers have suggested that the early use of IL-6 blockade as ‘add-on’ immunotherapy within the first 24 h (T1–T2) of onset demonstrates potential for improving the long-term outcome (Hosie et al., 2023).

### 4.3. Other therapies

#### 4.3.1. Vitamin cocktail therapy for mitochondrial dysfunction

Energy depletion resulting from decreased mitochondrial function is a potential cause of AE. Omata et al. (2016) conducted a study in which a vitamin cocktail consisting of vitamin B1, vitamin C, biotin, vitamin E, coenzyme Q10, and L-carnitine was administered within 24 h of AE diagnosis (within 48 h of onset, T1–T3) to patients with febrile convulsive status epilepticus. Patients with AE (including AESD and other AEs) who underwent vitamin cocktail treatment within 24 h of diagnosis exhibited better neurological outcomes at hospital discharge than those without vitamin cocktail treatment. Fukui et al. (2019) also reported that patients with AE who received treatment with vitamin B1, vitamin B6, and L-carnitine within 24 h of onset (T1–T2) had improved neurological outcomes (reduced developmental delay and lower incidence of post-encephalopathy epilepsy) compared with those without treatment. Furthermore, fewer patients in the vitamin-treated group were eventually diagnosed with AESD. Vitamin B6 acts as a coenzyme for glutamic acid decarboxylase and is believed to facilitate the conversion of glutamate to gamma-aminobutyric acid, which is a possible mechanism of action for its efficacy.

#### 4.3.2. KD

The KD is an established and effective non-pharmacological treatment for drug-resistant epilepsy. Recently, there has been an increasing number of reports on the efficacy of KD for SRSE. Additionally, the KD has been attempted as a means of seizure control in children with FIRES, a severe form of AE characterized by prolonged RSE triggered by an unknown fever. Nabbout et al. conducted a study involving nine KD-treated patients with FIRES (Nabbout et al., 2010). The KD was initiated between days 4 and 55 (T4), and SRSE was terminated within 2–4 days following the onset of ketonuria. Similarly, Singh et al. reported two cases of RSE wherein FIRES was successfully terminated using the KD, which was initiated on hospitalization days 3 and 13 (Singh et al., 2014). In addition to these studies, several other case series have reported the efficacy of KD therapy for RSE in patients with FIRES; however, these case series are not included in this review as they focused on status epilepticus treatment rather than encephalitis/encephalopathy treatment, and hence are beyond the scope of this review.

#### 4.3.3. Other treatment options

A case series report has evaluated the efficacy of dextromethorphan (an NMDA receptor antagonist and sigma-1 receptor agonist for AESD) in four patients with mild consciousness disturbance (Japan Coma Scale score < 30) occurring 24 h after prolonged (>15 min) FS (near-miss AESD) and four patients with AESD. In patients with near-miss AESD, dextromethorphan was started on day 2 or 3 (T3–T4), and no patient developed secondary seizures and AESD. In patients with AESD, dextromethorphan and cyclosporine were started when the secondary seizure was observed on days 4–6 (T4); three of the four patients had normal cognition after 1–3 years of follow-up, whereas one patient showed mild neurological sequelae (hyperactivity) after 3 years. The authors suggested that dextromethorphan and cyclosporine could be effective for the treatment and prevention of AESD (Matsuo et al., 2013). Sa et al. reported two cases of patients with FIRES treated with CMN-DBS and anakinra. CMN-DBS was performed on days 27 and 37 for SRSE control. Generalized seizures were terminated in both patients. One patient had no cognitive sequelae, whereas the other patient had severe sequelae and was in a vegetative state (Sa et al., 2019).

## 5. Conclusion

No randomized control trial has evaluated the AE treatment options; only 16 comparative studies with a control group and 14 case series reports with at least two cases are currently available. Disease conditions and the timing of treatment varied widely among the studies. Generally, the studies suggested that the treatments were effective in terms of ameliorating neurologic sequelae when administered within 12–24 h of AE onset. Moreover, treatments often targeted high-risk conditions prior to the diagnosis of AE syndromes. Nevertheless, KD and anakinra therapies may reduce seizure frequency and allow for weaning from barbiturates, even when administered weeks after onset in children with FIRES. This scoping review lays the groundwork for future prospective clinical trials.

## Author contributions

HN developed the main concepts and performed the scoping review and wrote the paper. MN performed the scoping review. All the authors reviewed and edited the manuscript and approved the final version.

## Funding

This study was supported by a Grant-in-Aid for Scientific Research (KAKENHI) from the Ministry of Education, Culture, Sports, Science, and Technology of Japan (Subject ID: 21K09047 to HN) and a Grant-in-Aid for Research on Measures for Intractable Diseases (21FC1005) from the Ministry of Health, Labor, and Welfare, Japan.

## Conflict of interest

The authors declare that the research was conducted in the absence of any commercial or financial relationships that could be construed as a potential conflict of interest.

## Publisher’s note

All claims expressed in this article are solely those of the authors and do not necessarily represent those of their affiliated organizations, or those of the publisher, the editors and the reviewers. Any product that may be evaluated in this article, or claim that may be made by its manufacturer, is not guaranteed or endorsed by the publisher.
